# Brain architecture in the terrestrial hermit crab *Coenobita clypeatus *(Anomura, Coenobitidae), a crustacean with a good aerial sense of smell

**DOI:** 10.1186/1471-2202-9-58

**Published:** 2008-06-30

**Authors:** Steffen Harzsch, Bill S Hansson

**Affiliations:** 1 Max Planck Institute for Chemical Ecology, Department of Evolutionary Neuroethology, Beutenberg Campus, Hans-Knöll-Str. 8, D-07745 Jena, Germany

## Abstract

**Background:**

During the evolutionary radiation of Crustacea, several lineages in this taxon convergently succeeded in meeting the physiological challenges connected to establishing a fully terrestrial life style. These physiological adaptations include the need for sensory organs of terrestrial species to function in air rather than in water. Previous behavioral and neuroethological studies have provided solid evidence that the land hermit crabs (Coenobitidae, Anomura) are a group of crustaceans that have evolved a good sense of aerial olfaction during the conquest of land. We wanted to study the central olfactory processing areas in the brains of these organisms and to that end analyzed the brain of *Coenobita clypeatus *(Herbst, 1791; Anomura, Coenobitidae), a fully terrestrial tropical hermit crab, by immunohistochemistry against synaptic proteins, serotonin, FMRFamide-related peptides, and glutamine synthetase.

**Results:**

The primary olfactory centers in this species dominate the brain and are composed of many elongate olfactory glomeruli. The secondary olfactory centers that receive an input from olfactory projection neurons are almost equally large as the olfactory lobes and are organized into parallel neuropil lamellae. The architecture of the optic neuropils and those areas associated with antenna two suggest that *C. clypeatus *has visual and mechanosensory skills that are comparable to those of marine Crustacea.

**Conclusion:**

In parallel to previous behavioral findings of a good sense of aerial olfaction in C. clypeatus, our results indicate that in fact their central olfactory pathway is most prominent, indicating that olfaction is a major sensory modality that these brains process. Interestingly, the secondary olfactory neuropils of insects, the mushroom bodies, also display a layered structure (vertical and medial lobes), superficially similar to the lamellae in the secondary olfactory centers of *C. clypeatus*. More detailed analyses with additional markers will be necessary to explore the question if these similarities have evolved convergently with the establishment of superb aerial olfactory abilities or if this design goes back to a shared principle in the common ancestor of Crustacea and Hexapoda.

## Background

**Figure 1:**
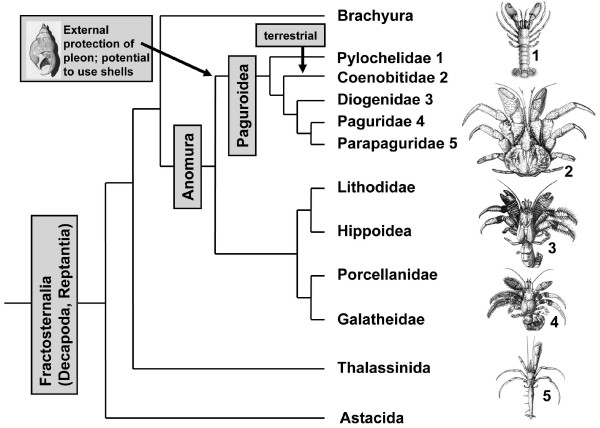
**Phylogenetic relationships of the reptantian taxa Astacida, Thalassinida, Anomura, and Brachyura according to**[44]. The Anomura and Brachyura are sister taxa. The phylogenetic relationships within the Anomura are a matter of debate. We decided to follow the hypothesis proposed by McLaughlin and co-workers [12]. The terrestrial Coenobitidae are a subtaxon of the Paguroidea, a group the members of which have evolved the potential to use molluscan shells as protection for their pleon. Images from [136].

Within Crustacea, at least five major lineages have succeeded in the transition from an aquatic to a fully terrestrial life style (reviews [1-4]). These include representatives of the Isopoda [5,6], of the Amphipoda [7-9], of the Astacida [10], of the Anomura [3,11], and of the Brachyura [3]. Within the Anomura, the Coenobitidae are a member of the Paguroidea (Fig. 1), a taxon the members of which have evolved the potential to protect the pleon with gastropod shells. The Coenobitidae comprise two genera that display a fully terrestrial life style [12]. They include 15 species of shell-carrying land hermit crabs (the genus *Coenobita*) and the robber or coconut crab *Birgus latro *(genus *Birgus*), the largest living land arthropod [11,13,14]. The early juvenile stages of this impressive creature, which as an adult can attain weights in excess of 5 kg, carry a shell, but with subsequent growth suitable shells are not available anymore so that the thorax and pleon harden for protection, as in other Crustacea [13,15]. Several other taxa within the Anomura, such as members of the Diogenidae and Porcellanidae (Fig. 1), also show terrestrial tendencies and occupy intertidal and mangrove habitats. However, only the Coenobitidae have developed terrestrial adaptations [16-18] that, apart from larval stages, allow them to permanently inhabit the supralitoral areas and small islands of tropical and subtropical maritime regions and to penetrate long distances inland [11,19].

The successful transition from marine to terrestrial life requires a number of physiological adaptations which are important for survival out of water. These are related e. g. to gas exchange, salt and water balance, nitrogenous excretion, thermoregulation, molting, and reproduction [1,2,4,11,16-18]. Concerning the nervous system, the sensory organs of terrestrial species must be able to function in the air rather than in the water. There is evidence that Coenobitidae have evolved good aerial visual abilities [11]. In olfaction, a transition from sea to land means that the stimulus changes from hydrophilic molecules in aqueous solution to mainly hydrophobic in the gaseous phase (discussed in [20]). Behavioral studies have provided evidence that these animals are very effective in detecting food from a distance and in responding to airborne odors, in short, that they have evolved a sense of distance olfaction that is behaviorally highly relevant for the animals [20-22]. The olfactory receptor neurons of crustaceans are associated with specialized structures on the first pair of antennae, the aesthetascs (reviews [23-25]). The aesthetascs of Coenobitidae are short and blunt and more similar to those of insects than to those of marine hermit crabs [20,26,27]. In robber crabs, they are confined to the ventral side of the primary flagella and arranged in ordered rows along a central groove. Contrary to marine crustaceans, they have an asymmetric profile with the protected side lined with a thick cuticle. The exposed side is covered with a thinner cuticle, a feature that most likely is necessary to enable the passage of odors [20]. Another clear distinction to marine crustaceans is that in the robber crab, the basal bodies and cilia segments are housed well inside the flagellum and are surrounded by a lymph space. Stensmyr et al. [20] interpreted these morphological features of the aesthetascs as adaptations to terrestrial conditions, more specifically, as mechanisms to minimize water evaporation while maintaining the ability to detect volatile odors from the gaseous phase. Terrestrial hermit crabs show flicking movements of their first antennae to maximize odor sampling, a strategy that is also applied by aquatic crustaceans [28,29]. In addition, Coenobitidae use their first antennae to touch and sample the ground [11], which suggests the presence of taste receptors.

The crustacean taxon that was undoubtedly most successful in the colonization of land is the Oniscoidea ("wood lice"), a subgroup of the Isopoda [2,6]. Within the Oniscoidea, the first pair of antennae is strongly reduced in size and instead the second pair of antennae seems to function as major sensory organs [6,30]. The tip of the second antennae bears a characteristic apical sensory cone that perceives mechanical and gustatory stimuli [30-33]. So far there is not any evidence that isopods use their second antennae for distance olfaction but for the desert isopod *Hemilepistus reaumuri *it was shown that contact chemosensors in their apical organs can detect polar, mainly non-volatile cuticle compounds of conspecifics and that this ability serves as the basis for a highly developed system of kin recognition (review [34]). The animals probe each other with the apical organs that react to carbonic acid, amines, sugar, fatty acids, amino acids and other substances [32,35]. It has previously been noted that, coinciding with the minute size of their first pair of antennae, in Oniscoidea the primary olfactory centres in the deutocerebrum, the olfactory lobes, are reduced in size [36-38]. We carried out a set of immunohistochemical studies on the brain architecture of several marine and terrestrial Isopoda (Harzsch and Hansson, unpublished data). Our study provided supportive evidence that the Oniscoidea have completely abandoned their olfactory lobes in response to the colonization of land. This suggests that, contrary to the Coenobitidae, the deutocerebral olfactory pathway does not play a significant role for aerial olfaction in the terrestrial isopods. It would appear that it is not trivial for any crustacean to establish an aerial sense of olfaction during the transition from sea to land.

Along these lines of arguments, the present study sets out to explore the architecture of the central olfactory processing areas in representatives of the Coenobitidae, for which the sense of smell has been proven to play an important role.

The architecture of the brain in land hermit crabs is poorly understood as is the nervous system architecture of Anomura in general [39]. So far, concerted studies on the brain morphology in this group have not been conducted, yet more or less incidental reports are available for representatives of the aquatic anomuran genera *Pagurus *(Paguroidea; see Fig. 1), and *Petrolisthes *(Porcellanidae) as well as *Munida quadrispina *(Galatheidae), and the fully terrestrial Coenobitidae [40-43]. The Thalassinida and Brachyura are the closest relatives to the Anomura (Fig. 1; [44]). The brains of *Calocaris *and the semi aquatic *Callianassa*, both members of the Thalassinida, and of several representatives of the brachyuran crabs have been analyzed in greater depths than those of the Anomura [40,43,45-47], including developmental studies on the larval brachyuran brain [48-51]. Concerning the terrestrial Anomura, a study by Sandeman and co-workers [40] on the robber crab *Birgus latro *had provided preliminary evidence for the presence of extremely large olfactory lobes in this species. Beltz and coworkers [42] conducted a numerical analysis on the olfactory glomeruli in 17 species of reptantian crustaceans including *Coenobita clypeatus*. With 800 glomeruli this species ranked second to the Achelata (clawless lobsters) as far as glomerular numbers were concerned. Furthermore, this terrestrial hermit crab ranked third concerning olfactory lobe volume and glomerular volume [42]. Taken together, these previous neuroanatomical studies in concert with the available behavioral reports indicate the presence of sophisticated olfactory systems in members of the Coenobitidae so that we decided to explore brain morphology in this group more closely. Specifically, we wanted to know if, other than aspects related glomerular numbers, their brains show any modifications such as deletions or addition of neuropil structures that compared to other aquatic Crustacea may be interpreted as adaptations to the terrestrial life style. Are the general brain layout of the Coenobitidae and the relative proportion of brain neuropils similar to that of other malacostracan Crustacea? Or have additional structures and different neuropil architectures evolved? To answer these questions, we analyzed the brain of *Coenobita clypeatus *(Herbst, 1791; Anomura, Coenobitidae), a fully terrestrial tropical species that penetrates long distances inland [11] by immunohistochemistry against synaptic proteins, serotonin, FMRFamide-related peptides, and glutamine synthetase. These markers were chosen to provide both, a general overview over the brain layout and a more detailed insight into the branching patterns of certain classes of neurons. Our results indicate that in fact their central olfactory pathway is most prominent, indicating that olfaction is a major sensory modality that these brains process.

## Results

The data reported here stem from three sets of triple labeling experiments i.e. combinations of markers (see material and methods):

1: synapsin + actin + nuclei;

2: RFamide + synapsin + nuclei;

3: glutamine synthetase + serotonin + nuclei.

In the figures, we use color-coded abbreviations to identify the markers:

SYN: synapsin immunoreactivity

RF: RFamide-like immunoreactivity

GS: glutamine synthetase-like immunoreactivity

5HT: serotonin-immunolocalization

ACT: phalloidin histochemistry to label actin

NUC: nuclear counter stain with bisbenzimide

**Figure 2:**
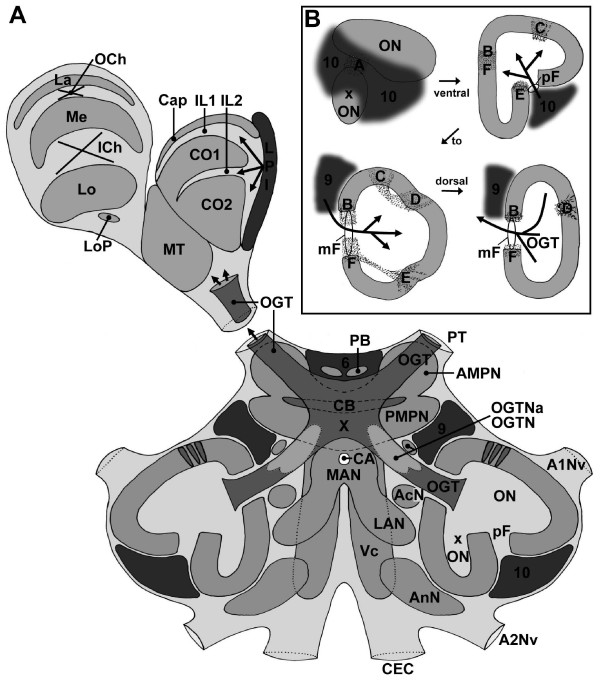
**A: Idealized schematic drawing of the *C. clypeatus *brain (dorsal view) compiled from ca. 4–5 successive sections (80 μm) of several animals at a mid horizontal level.** B: Schematic cartoons of ventral to dorsal sections of the main olfactory lobe (ON) and the side olfactory lobe (xON) to show the localization of the median (mF) and posterior foramina (pF) and the patches of non-columnar neuropil (dotted areas labeled with letters A-F). Arrows labeled 9 and 10 show the input of olfactory interneurons. The arrow labeled OGT shows the exit of the olfactory globular tract. Abbreviations: 6, 9, 10 cell clusters 6, 9, 10, A1Nv nerve of antenna 1, A2Nv nerve of Antenna 2, AcN acessory lobe/neuropil, AMPN anterior medial protocerebral neuropil, AnN antenna 2 neuropil, CA cerebral artery, Cap cap neuropil of the hemiellipsoid body, CB central body, CEC circumesophageal connectives, CO1, CO2 core neuropils 1 and 2 of the hemiellipsoid body, ICh inner optic chiasm, IL1, IL2 intermediate layers 1 and 2 of the hemiellipsoid body, La Lamina (lamina ganglionaris), LAN lateral antenna 1 neuropil, Lo Lobula (medulla interna), LoP Lobula "plate", LPI lateral protocerebral interneurons, MAN median antenna 1 neuropil, Me Medulla (medulla externa), mF median foramen, MT Medulla terminalis, OCh outer optic chiasm, OGT olfactory globular tract, OGTN olfactory globular tract neuropil, OGTNa accessory olfactory globular tract neuropil, ON olfactory lobe/neuropil, PB protocerebral bridge, pF posterior foramen, PMPN posterior medial protocerebral neuropil, PT protocerebral tract, VC ventral neuropil column, X chiasm of the olfactory globular tract.

**Figure 3:**
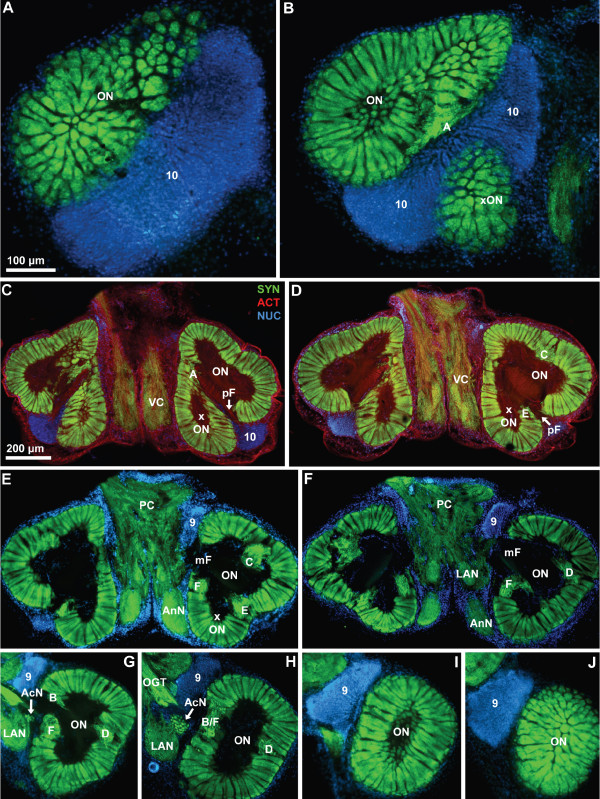
**Low power views of a ventral to dorsal section series featuring anti-synapsin immunohistochemistry (SYN; green) with actin (ACT; red) and nuclear (NUC; blue) counter stains; conventional fluorescence combined with the Apotome structured illumination technique for optical sectioning.** In the first two most ventral slices A and B, the left hemisphere is shown). Numbers 9 and 10 identify cell clusters. Letters A to F identify the non-columnar olfactory neuropils. Other abbreviations: AcN accessory lobe/neuropil, AMPN anterior medial protocerebral neuropil, AnN antenna 2 neuropil, LAN lateral antenna 1 neuropil, mF median foramen, OGT olfactory globular tract, ON olfactory lobe/neuropil, PC protocerebrum, pF posterior foramen, PMPN posterior medial protocerebral neuropil, xON side olfactory lobe/neuropil, VC ventral neuropil column.

**Figure 4:**
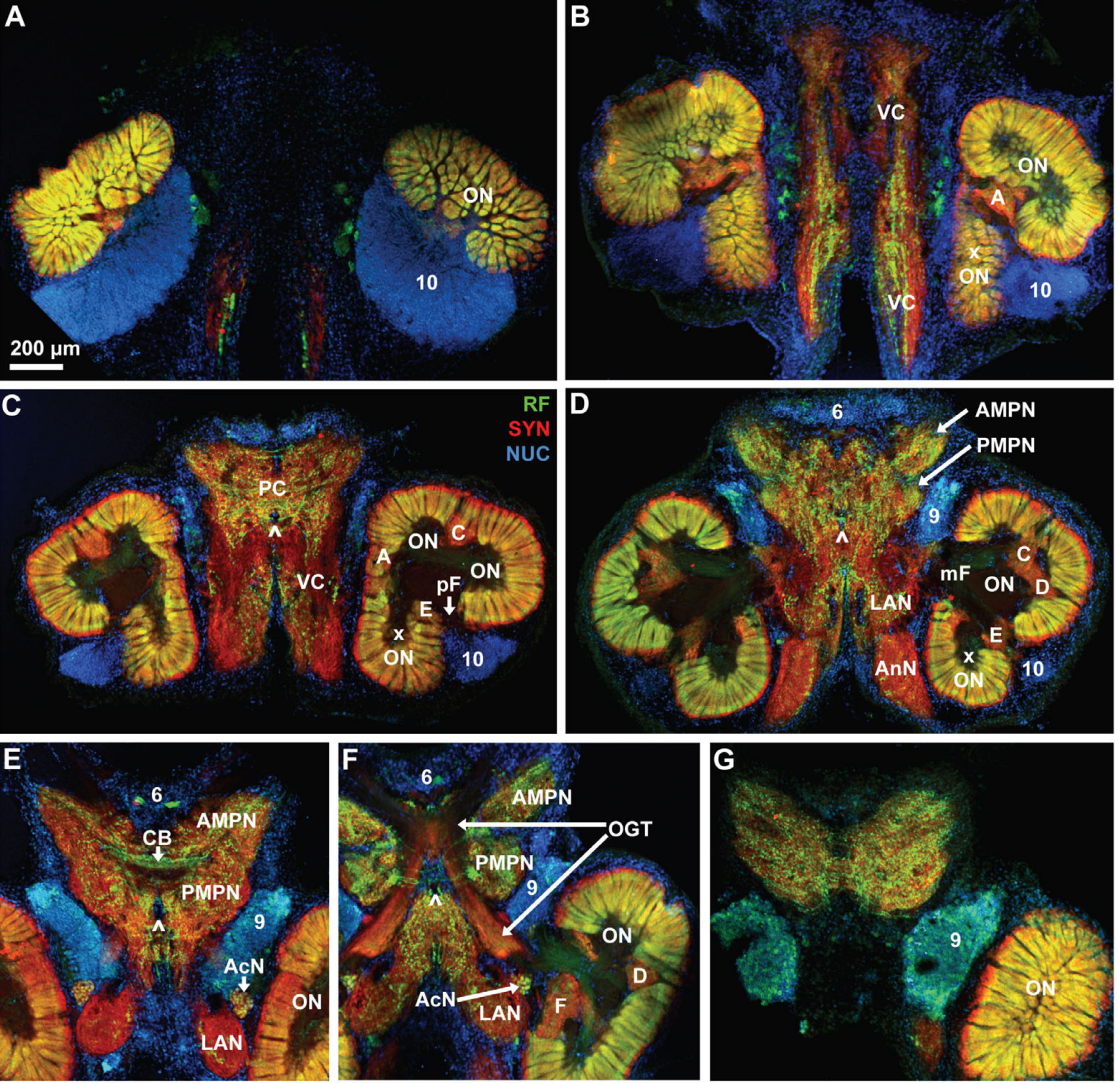
**Low power views of a dorsal to ventral series of vibratome sections triple labeled for synapsin immunoreactivity (SYN; red), RFamide-like immunoreactivity (RF; green), and the nuclear marker (NUC; blue); conventional fluorescence combined with the Apotome structured illumination technique for optical sectioning.** Note that section E is from another animal and inserted here because it provides a better view of the central body (CB). Section E is not perfectly horizontal so that the medial foramina of the olfactory lobes are not visible here. The arrows in C to F point to the cerebral artery that pierces the brain. Numbers 6, 9, 10 identify cell clusters. Letters A to F identify the non-columnar olfactory neuropils. Other abbreviations: AcN accessory lobe/neuropil, AMPN anterior medial protocerebral neuropil, AnN antenna 2 neuropil, LAN lateral antenna 1 neuropil, mF median foramen, OGT olfactory globular tract, ON olfactory lobe/neuropil, PC protocerebrum, pF posterior foramen, PMPN posterior medial protocerebral neuropil, xON side olfactory lobe/neuropil, VC ventral neuropil column.

**Figure 5:**
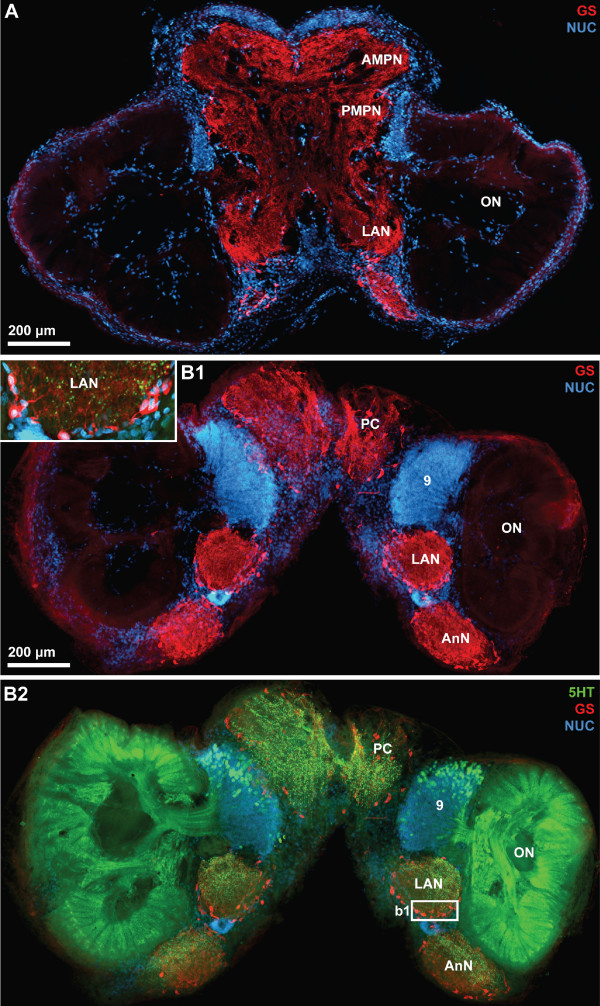
**Low power views of horizontal vibratome sections triple labeled for serotonin immunoreactivity (5HT; green), glutamine synthetase-like immunoreactivity (GS; red), and the nuclear marker (NUC; blue); conventional fluorescence combined with the Apotome structured illumination technique for optical sectioning.** A is more ventral than B1 and B2. B1 and B2 show different labels in the same section. Strong glutamine synthetase-like immunoreactivity is present in all brain neuropils except the olfactory lobes. The inset in B1 shows a higher magnification of the boxed region in B2. Putative ensheathing glia cells are arranged around the periphery of the lateral antenna 1 neuropil. Number 9 identifies cell cluster (9) that houses local olfactory interneurons that are serotonergic and innervate the olfactory lobes Other abbreviations: AMPN anterior medial protocerebral neuropil, AnN antenna 2 neuropil, LAN lateral antenna 1 neuropil, ON olfactory lobe/neuropil, PC protocerebrum, PMPN posterior medial protocerebral neuropil.

The experiments provided a consistent picture of the gross brain anatomy so that we were able to compile a schematic drawing of the *C. clypeatus *brain (Fig. 2). We will first give an overview about the labeling pattern with these three marker sets (Figs. 3, 4, 5) and then will describe specific brain structures in more detail, from the protocerebrum across the deuto- and tritocerebrum to end with the eyestalk neuropils. We determined the sex of the specimens that were studied according to their pleopod morphology, but did not encounter any sex-specific differences of their brains. Most of the brain structures (except for example the central body) are bilaterally paired. For simplicity we will describe only one brain hemisphere (mostly the right side) in the understanding that mirror symmetrical structures are present in the contralateral hemisphere.

### Overview over the *C. clypeatus *brain

Fig. 3 shows a ventral to dorsal section series featuring anti-synapsin immunohistochemistry (green) with actin (red) and nuclear (blue) counter stains. In the two most ventral slices (Fig. 3A, B; the left hemisphere is shown), tangential sections of the olfactory neuropil or olfactory lobe (ON) can be seen. In Crustacea the ON receives afferent chemosensory input from olfactory receptor neurons on the paired first antennae. The olfactory neuropil is composed of numerous column-like structures with strong synapsin-immunoreactivity (SYNir), the "olfactory" glomeruli. Despite their columnar shape we will refer to these neuropil elements as glomeruli since this term is well introduced in the literature. In cross sections (Fig. 3A), these structures appear as round profiles whereas in the following sections it becomes apparent that the glomeruli are arranged parallel to each other around the periphery of the lobe. The centre of the lobe is devoid of SYNir, yet actin labeling shows that this core is filled with bundles of fibrous material (Fig. 3C, D). Histochemical labeling of cell nuclei reveals a densely packed cluster with hundreds if not thousands of neuronal somata to be associated with the olfactory lobe. This cluster most probably corresponds to cluster (10), which is known to house olfactory projection neurons in other malacostracan Crustacea [52]. In *C. clypeatus*, cluster (10) is located medially and posteriorly to the ON in the most ventral aspect but also extends more dorsally, where it wraps around the posterior part of the ON (Fig. 3C). In subsequent sections, a side lobe of the olfactory neuropil (xON) becomes visible that in the more ventral sections seems to be separate from the main ON (Fig. 3B). Proceeding further dorsally, however, it becomes apparent that this side lobe is connected to the main ON (Fig. 3C–F). Medial to the ON, SYNir reveals a horizontal column of loose, unstructured neuropil that extends in an anterior-posterior direction, the ventral neuropil column (VC; Fig. 3D). Further dorsally (Fig. 3E–H), two compact, medially situated neuropils become visible displaying strong SYNir: the lateral antenna 1 neuropil (LAN), and the antenna 2 neuropil (AnN). At this level, in the protocerebrum (PC), unstructured immunolabelled neuropil is visible. Between the protocerebrum and the anterior part of the ON, a second compact cell cluster with densely packed nuclei is visible. This is most likely cell cluster (9) that houses local olfactory interneurons [52]. This cell cluster extends through at least five 80 μm sections and once again houses hundreds or thousands of neurons (Fig. 3F–J). In other sections, nuclear labeling shows that the brain is surrounded by a thick layer of cell nuclei, but we could not differentiate which of these belong to the perineurium and which may be neurons. The accessory lobe (AcN) is an assemblage of small SYNir glomeruli and is located medially to the olfactory lobe close to the point where the olfactory globular tract (OGT) emerges from the latter (Fig. 3H). The ON clearly is the dominating structure of the *C. clypeatus *brain and dorso-ventrally stretches through the entire section series. In the most dorsal section, once again cross sections of the radially arranged olfactory glomeruli are visible (Fig. 3J).

Fig. 4 shows ventral to dorsal section series of another specimen that was processed for anti-synapsin immunohistochemistry (red), RFamide-like immunohistochemistry (green) and a nuclear counter stain (blue). This series reveals a few additional structures compared to Fig. 3 but the general arrangement and size of the main neuropils is similar in this and several other specimens that we examined. The orange color of the olfactory lobes indicates that in the olfactory glomeruli, SYNir and RFamide-like immunoreactivity (RFir) are mostly co-localized (Fig. 4A, B). In the middle of the brain, however, where the glomeruli are sectioned longitudinally, it becomes clear that the cap region of the glomeruli shows only SYNir (red) but not RFir (green). In the ventral neuropil column, RFir fibers are embedded and RFir somata are located between this column and the ONs (Fig. 4B, C). The protocerebrum is filled with a loose network of RFir fibers. In this section series, the subdivision of the protocerebral neuropil in an anterior and a posterior component, that is so typical of decapod crustaceans [52], is visible. These are the anterior (AMPN) and posterior medial protocerebral neuropils (PMPN; Fig. 4D–F). The central body (CB) is a transverse, unpaired protocerebral neuropil that extends across the midline, is embedded between the two aforementioned protocerebral compartments, and displays strong RFir (Fig. 4E). A thick, paired fiber bundle, the olfactory globular tract, leaves the olfactory lobes in a medial direction and surprisingly seems to display both RFir and SYNir (Fig. 4F). The left and right portions of this tract touch each other at the midline, slightly above the central body, where they form a characteristic chiasm. The two bundles then separate again to veer antero-laterally and exit the medial brain by joining the protocerebral tract to target the lateral protocerebrum in the eyestalks (see below). Cell cluster (6) is situated anteriorly between the two arms of the protocerebral tract. The accessory lobe, being situated close to the origin of the olfactory globular tract displays both RFir and SYNir. Medially, a block of diffuse neuropil, the median antenna 1 neuropil (MAN) is embedded between the two arms of the olfactory globular tract (Fig. 4F). In the most dorsal section (Fig. 4G) it becomes apparent that many cell somata in cluster (9) display strong RFir.

**Figure 6:**
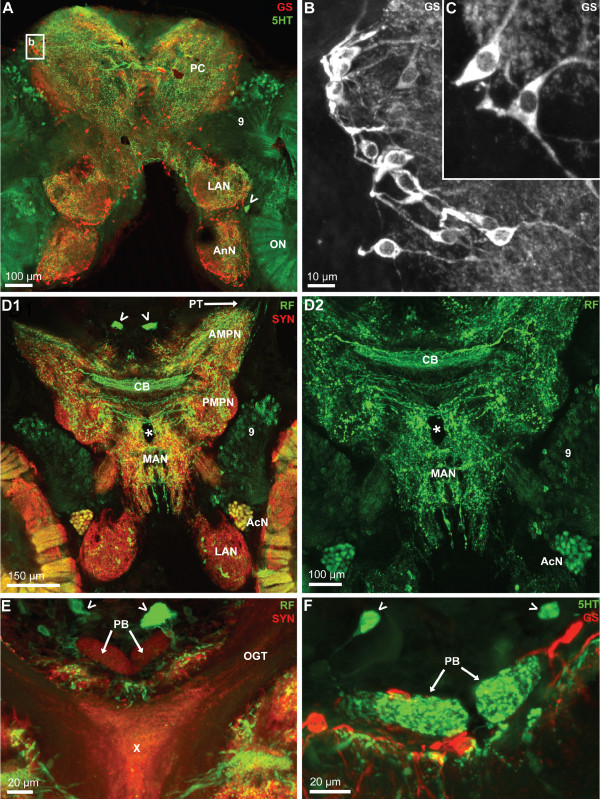
**A-C: Higher magnifications of the protocerebrum and median deutocerebrum.** Horizontal vibratome sections double labeled for serotonin immunoreactivity (5HT; green) and glutamine synthetase-like immunoreactivity (GS; red); confocal laser scan microscopy. The boxed area in A is shown in a higher magnification in C. The neuropils surrounded by GSir cells display strong immunolabelling in the neuropil core. The bi- or tripolar GSir cells, presumably ensheathing glia cells, extend processes into the neuropil. The arrow in A identifies a large serotonergic neuron. Many more serotonergic somata are located in cell cluster (9). D1, D2: Double labeled sections showing synapsin immunoreactivity (SYN; red) and RFamide-like immunoreactivity (RF; green); confocal laser scan microscopy. In D2, only the green channel is visualized at a slightly higher magnification to show the central body (CB). The arrowheads in D1 identify peptidergic cell somata. E, F: The protocerebral bridge (PB) displays synapsin immunoreactivity (E) and serotonin immunoreactivity (F; confocal laser scan microscopy). The arrowheads identify cell somata within cell cluster (6). The X labels a synaptic region in the chiasm of the olfactory globular tract. Abbreviations: 9 cell cluster (9), AcN accessory lobe/neuropil, AMPN anterior medial protocerebral neuropil, AnN antenna 2 neuropil, LAN lateral antenna 1 neuropil, MAN median antenna 1 neuropil, OGT olfactory globular tract, ON olfactory lobe/neuropil, PC protocerebrum, PMPN posterior medial protocerebral neuropil, PT protocerebral tract.

Fig. 5 shows two horizontal sections of another specimen that was processed for anti-serotonin immunohistochemistry (green), glutamine synthetase-like immunohistochemistry (red) and a nuclear counter stain (blue). Cell somata with strong glutamine synthetase-like immunoreactivity (GSir) surround all brain neuropils with the exception of the ONs. Within the ON, there is a very faint and diffuse labeling. We were unable to decide if it represents a specific signal or just unspecific background labeling. A tissue layer displaying weak GSir, presumably the perineurium [53], surrounds the entire brain (Fig. 5B). Within the cell clusters known to comprise neuronal cell bodies such as cluster (9), typically very few or no GSir somata were present (Fig. 5B1, 6A). Those neuropils surrounded by GSir cells also display strong immunolabelling in the neuropil core (Fig. 5A, B1, 6A). At higher magnification, the GSir cells at the periphery of the neuropil can be seen to extend processes into the neuropil (Fig. 5B1 inset). These cells are typically bi- or tripolar (Fig. 6B, C). Comparing the labeling pattern observed here to other studies on crustacean glia cells [54-56] suggests that GSir in *C. clypeatus *is strongly localized in a certain type of glia cells, the ensheathing glia [53] but not in neurons. Serotonin-immunoreactivity (5HTir) is widespread throughout the *C. clypeatus *brain. The protocerebrum, the lateral antenna 1 neuropil, as well as the antenna 2 neuropil display strong 5HTir (Fig. 5B2, 6A). A population of serotonergic olfactory interneurons with somata within cell cluster (9) gives rise to a strong innervation of the ON.

### Protocerebrum

**Figure 7:**
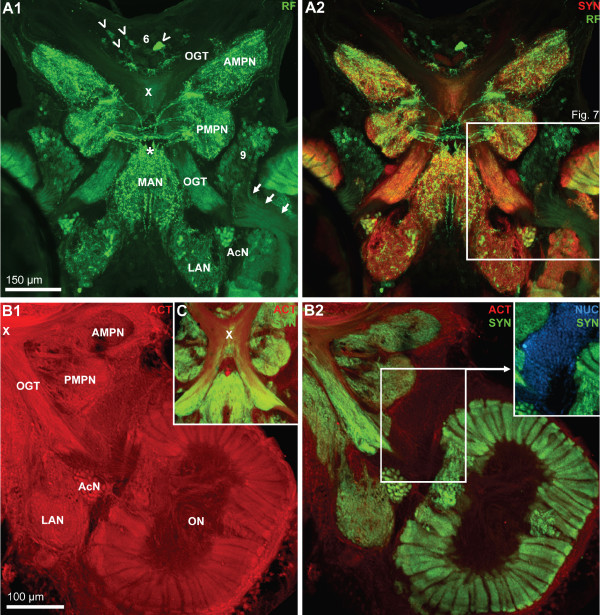
**A1, A2: A horizontal section double labeled for synapsin immunoreactivity (SYN; red) and RFamide-like immunoreactivity (RF; green) to show the course of the olfactory globular tract (OGT); confocal laser scan microscopy.** The X labels the chiasm of the olfactory globular tract. Arrowheads identify peptidergic somata in the anteriorly located cell cluster (6). Arrows point towards a bundle of neurites from cluster (9) olfactory interneurons that penetrate into the olfactory lobe. The cerebral artery is labeled with an asterisk. The boxed area in A2 is shown in a higher magnification in Fig. 2. B1, B2, C: Triple labeled section showing actin histochemistry (ACT; red), Synapsin immunoreactivity (SYN; green), and localization of nuclei (NUC; blue) to show the course of the olfactory globular tract (OGT); conventional fluorescence combined with the Apotome structured illumination technique for optical sectioning. The X labels the chiasm of the olfactory globular tract. A2 shows the red channel only, and B2 and C the red and green channel. The inset in B2 shows the green and the blue channel. Abbreviations: 6 cell cluster (6), AcN accessory lobe/neuropil, AMPN anterior medial protocerebral neuropil, LAN lateral antenna 1 neuropil, MAN median antenna 1 neuropil, OGT olfactory globular tract, ON olfactory lobe/neuropil, PMPN posterior medial protocerebral neuropil.

The protocerebrum can be subdivided into an anterior and a posterior component, the anterior (AMPN) and posterior medial protocerebral neuropils (PMPN; Fig. 6D1, 7A1, 7B1). This subdivision is most obvious in the middle of the section series (Fig. 4D–F), at the level of the olfactory globular tract chiasm (see below), whereas more ventrally (Fig. 4C) and more dorsally (Fig. 4G, 5B1, B2, 6A) such a distinction is not possible. In the protocerebral tract that links the anterior median protocerebral neuropil to the lateral protocerebrum, RFir fibers are present (Fig. 6D1). Anteriorly, the protocerebral neuropil is adjoined by the cell cluster (6) in which neuronal somata with both RFir (arrowheads in Fig. 6D1, E, 7A1) and 5HTir (arrowheads in Fig. 6F) are located. At the interface between the anterior (AMPN) and posterior medial protocerebral neuropils, a transverse, unpaired neuropil extends across the midline, the central body (CB; Fig. 6D1, D2). The central body is innervated by a dense plexus of fine RFir (Fig. 6D2) and 5HTir fibers (data not shown). Several thick RFir commissural fiber bundles accompany the central body posteriorly (Fig. 6D1, D2) and dorsally (Fig. 7A1). Behind these commissural fibers, the cerebral artery pierces the brain in a dorso-ventral direction (arrowheads in Fig. 4C–F, asterisk in Fig. 6D1, D2). The protocerebral bridge is located anteriorly and slightly dorsal to the central body at the level of the olfactory globular tract chiasm (Fig. 6E, F). Its bilaterally symmetrical neuropil compartments adjoin each other at the midline and show positive SYNir and 5HTir but not any RFir.

### The olfactory globular tract

**Figure 8:**
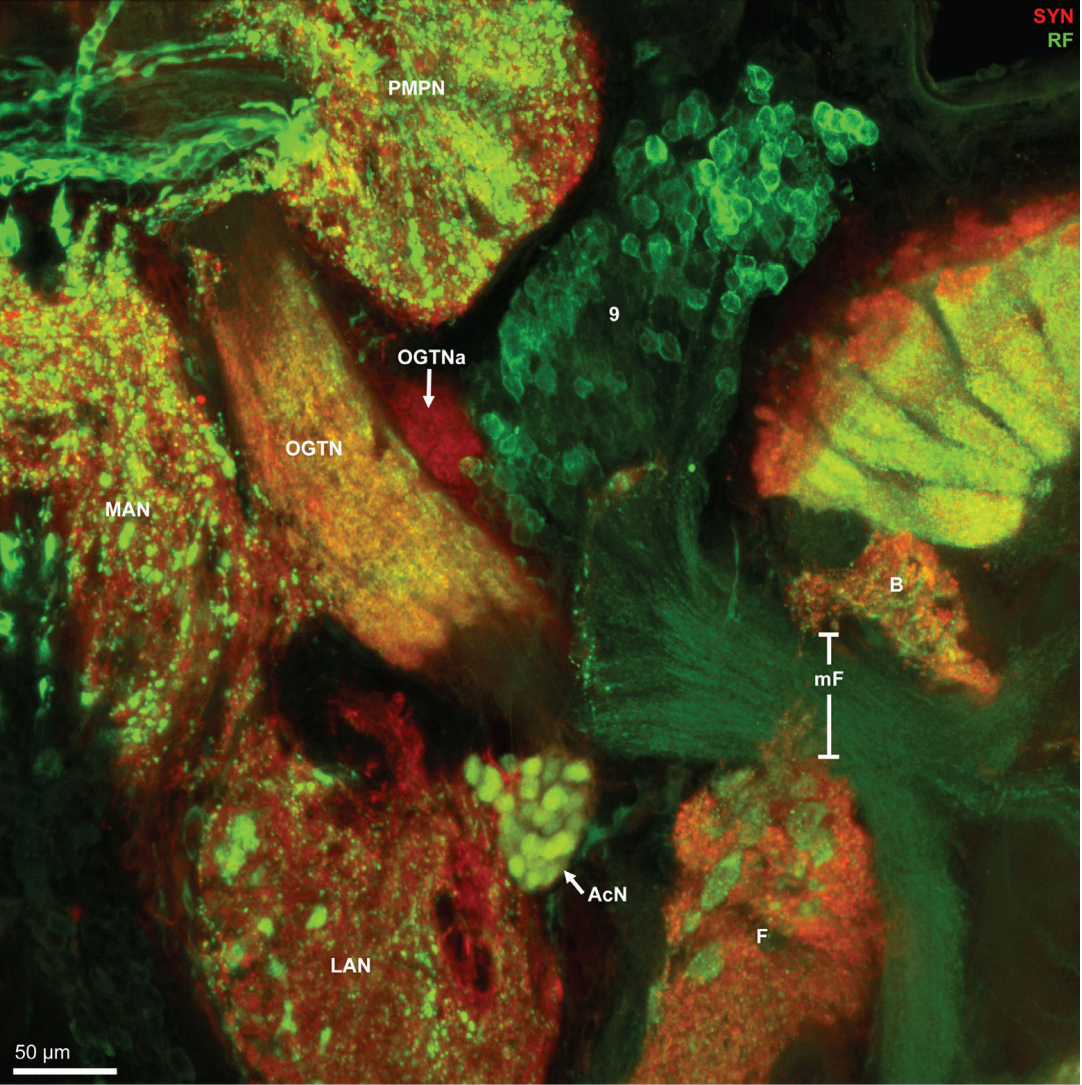
**Higher magnification of the section shown in Fig. 6A2, synapsin immunoreactivity (SYN; red) and RFamide-like immunoreactivity (RF; green); confocal laser scan microscopy.** Peptidergic olfactory interneurons the somata of which are located in cell cluster (9) project their neurites through the median foramen (mF) into the olfactory lobe. In the medial antenna 1 neuropil, the lateral antenna 1 neuropil, and the non-columnar olfactory neuropils F, large, round RFamidergic profiles (putative peptidergic release sites) are embedded whereas in the posterior median protocerebral neuropil (PMPN) finer profiles are present. The glomeruli in the accessory neuropil (AcN) show both, synapsin- and RFamide-like immunoreactivity. Within the olfactory globular tract (OGT), a synaptic neuropil region is embedded that also shows RFamide-like immunoreactivity. The accessory olfactory globular tract neuropil (OGTNa) that is associated with the olfactory globular tract, shows synapsin immunoreactivity but not any peptidergic labeling.

The olfactory globular tract links the olfactory and accessory neuropils to the lateral protocerebrum. In Decapoda, this tract is formed by the axons of the projection neurons with their somata in cell cluster (10) and this tract represents the major output pathway of the olfactory system (reviews [52,57,58]). In the brain of *C. clypeatus*, actin labeling revealed the course of this tract (Fig. 7B1, C, 13A). After emerging from the olfactory lobes it courses antero-medially to meet its contralateral counterpart in a chiasm slightly dorsal to the central body (the chiasm is identified by the X in Fig. 6E, 7A–C). Its two arms then separate again to proceed antero-laterally to leave the brain via the protocerebral tract and to target the lateral protocerebrum (see below). Surprisingly, synapsin labeling provided evidence for synaptic material to be associated with two regions of the olfactory globular tract, namely a region within the chiasm (Fig. 6E), and a long section between the olfactory lobe and the chiasm (Fig. 3H, 7A2, B2, C; see also Fig. 13A). This section of the olfactory globular tract also displays strong RFir (Fig. 7A1). At a higher magnification it becomes clear that the regions of SYNir and RFir in this stretch of the olfactory globular tract almost completely overlap (Fig. 8) suggesting the presence of synapses with RFamide-like neuropeptides to be associated with the tract. This synaptic region may correspond to the olfactory globular tract neuropil as found in other Decapoda [52]. The olfactory globular tract is laterally accompanied by a small, spherical neuropil, the accessory olfactory globular tract neuropil (OGTNa) that displays SYNir but not RFir (Fig. 8, 13A).

### Deutocerebrum: the olfactory neuropils

**Figure 9:**
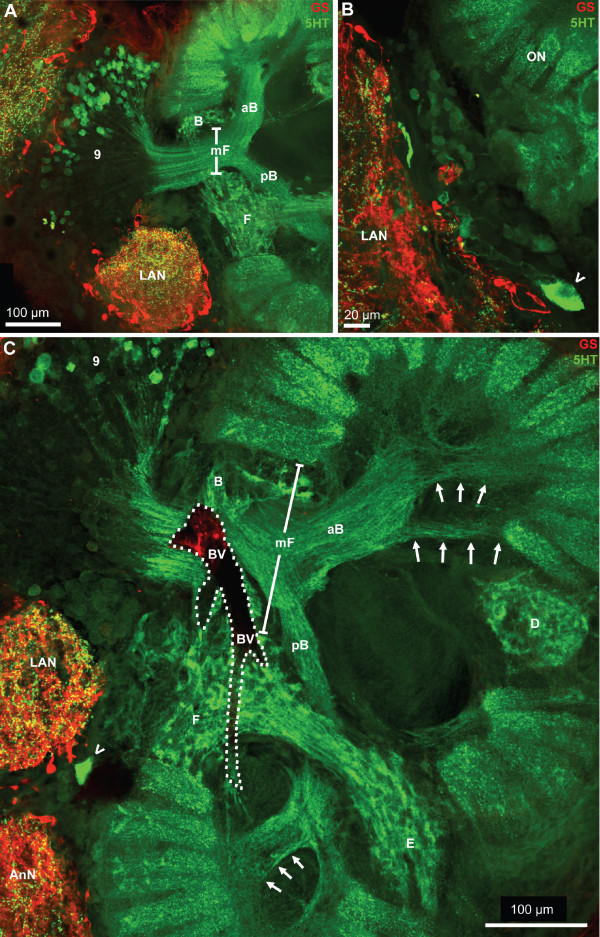
**A-C: Serotonergic innervation of the olfactory lobes.** Double labeled sections showing glutamine synthetase-like immunoreactivity (GS; red) and serotonin immunoreactivity (5HT; green); confocal laser scan microscopy. A large population of serotonergic local interneurons in cell cluster (9) sends neurites into the core of the olfactory lobe (ON) by passing the median foramen (mF). This foramen is flanked by the non-columnar olfactory neuropils B and F the latter of which is connected by a neuropil bridge to neuropil E. The thick neurite bundles from cluster (9) interneurons, after entering the olfactory lobe, split into a large anterior (aB) and posterior bundle (pB). These bundles then branch out into finer bundles the fibers in which approach the proximal part of the olfactory glomeruli (arrows in C). Between the olfactory neuropil (ON) and the lateral antenna 1 neuropil (LAN), the soma of at least one large serotonergic neuron is located (arrowheads in B, C) but the axonal projection could not be traced. The dotted line in C encircles a blood vessel (BV).

Two large clusters of olfactory interneurons are associated with the olfactory neuropils, cell cluster (10) with projection neuron somata, and cluster (9) that houses local olfactory interneurons (Fig. 3, 4). None of the projection neurons in cluster (10) displays 5HTir or RFir. Yet, in cluster (9) large populations of local interneurons are present that display strong 5HTir (Fig. 5B2, 9A, C) or RFir (Fig. 6D1, D2, 7A1, A2, 8) and the neurites of which extend into the core of the ON. We did not analyze if some cluster (9) neurons co-localize serotonin and RFamide. Between the ON and the lateral antenna 1 neuropil, the soma of at least one large serotonergic neuron is located (arrowheads in Fig. 6A, 9B, C) but the axonal projection could not be traced. Bundles of fine neurites of the cluster (9) interneurons enter the ONs from the medial side in a thick bundle that then branches out into finer bundles (Fig. 8, 9, 11B, C1, C2), in which fibers approach the proximal part of the olfactory glomeruli. In specimens processed for 5HTir, a large anterior (aB) and posterior bundle (pB) of cluster (9) neurites can be distinguished, where fibers spread out towards the bases of glomeruli (arrows in Fig. 9C).

**Figure 10:**
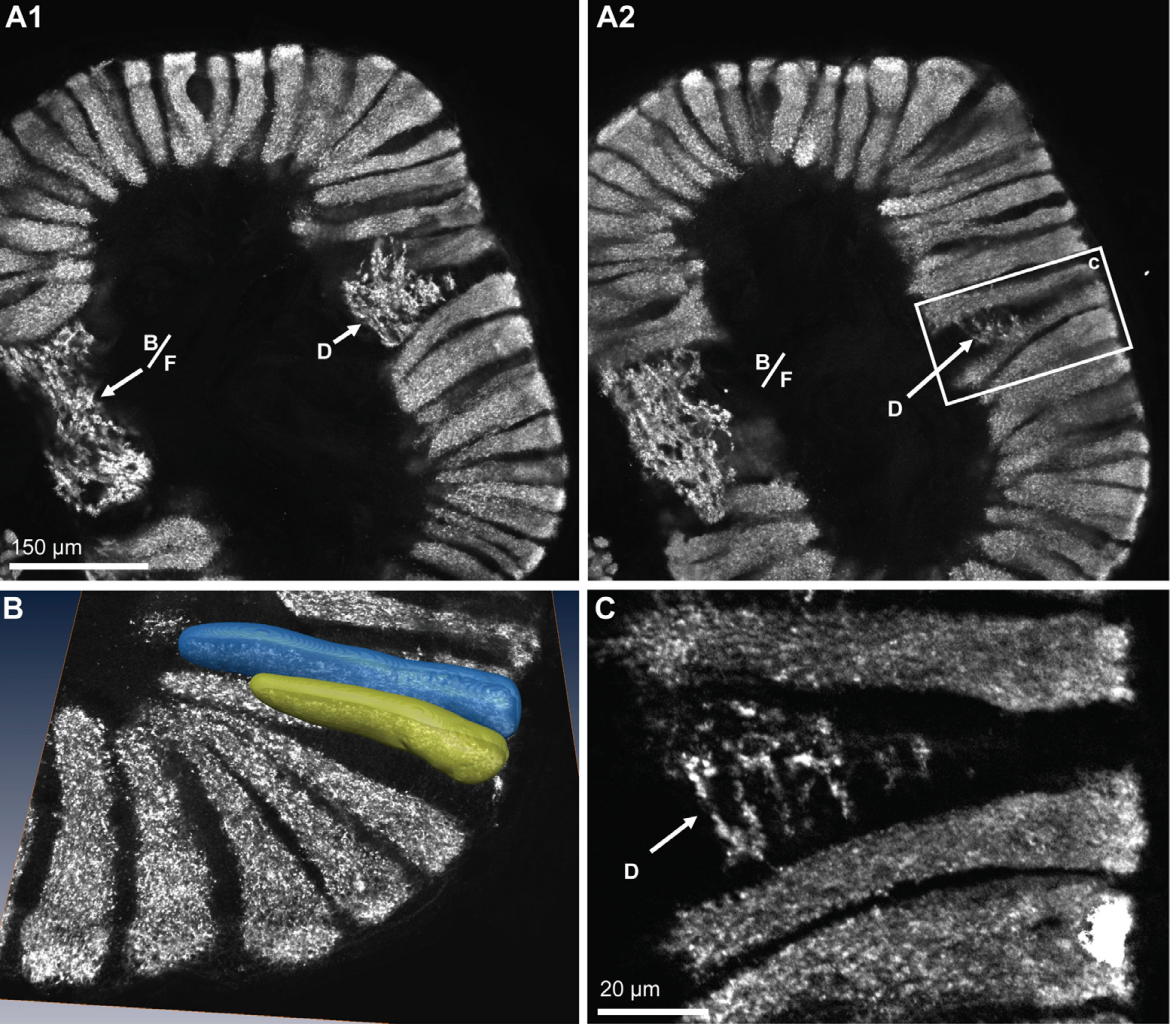
**Immunolocalization of synapsin in the olfactory lobes shows that synaptic neuropil is confined to the olfactory glomeruli and the non-columnar olfactory neuropil (letters B, D, F).** A1, A2: two optical sections (Apotome structured illumination technique) from different levels of one vibratome section. The non-columnar olfactory neuropils B and F are merged here and line the dorsal part of the median foramen. The non-columnar olfactory neuropil D is embedded within the olfactory glomeruli. The boxed area in A2 is shown in a higher magnification in C. This optical section (Apotome structured illumination technique) shows that there is not any overlap between the synaptic regions of both types of olfactory neuropils (columnar *versus *non-columnar). D shows a surface reconstructions obtained from a z-series of confocal images that were directly loaded into Amira and processed for semiautomatic segmentation using Amira's "wrap" module.

**Figure 11:**
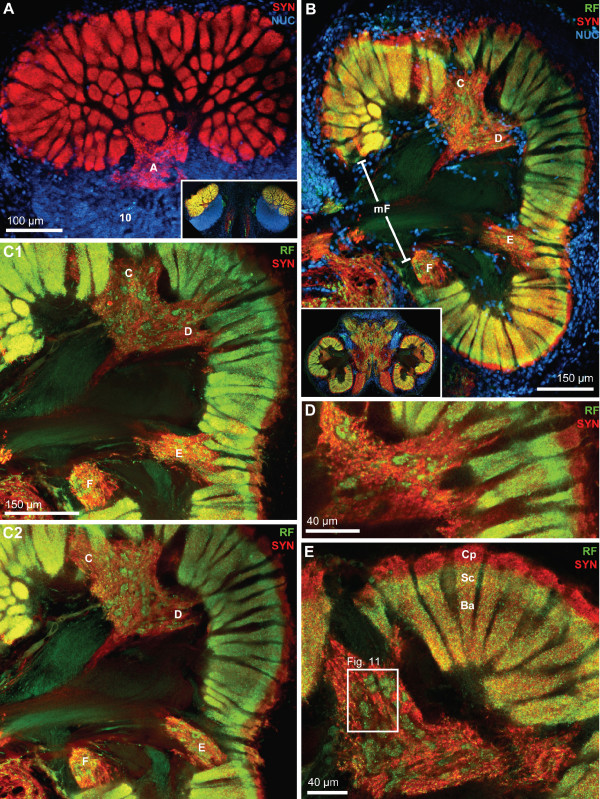
**Horizontal vibratome sections to show the localization of the non-columnar olfactory neuropils and the regionalization of the olfactory glomeruli.** Double labeling (C1-E) for synapsin immunoreactivity (SYN; red) and RFamide-like immunoreactivity (RF; green), or triple labeling for these two substances plus the nuclear marker (NUC; A, B); conventional fluorescence combined with the Apotome structured illumination technique for optical sectioning (A, B) and confocal laser scan microscopy (C1-E). A: superficial, tangential section of the right olfactory lobe (compare inset and Fig. 3D) to show the localization of non-columnar olfactory neuropil A. Cell cluster (10) is also visible. B: Overview over the arrangement of non-columnar olfactory neuropils C-F and the medial foramen (mF). The inset shows a low power view of this section (compare Fig. 3D). C1, C2: these two images show the non-columnar olfactory neuropils in B at a higher magnification. The images are from a stack of 30 optical sections covering 22 μm in the z direction. Image C1 is a projection of 6 sections covering z = 9.1 μm to z = 12.9 μm. Image C2 is a projection of 6 sections covering z = 18.2 μm to z = 21.2 μm. The non-columnar olfactory neuropil C is merged with D. Both are closely associated with the proximal bases of olfactory glomeruli and in some places even seem to overlap with the glomeruli (Fig. 9A1, 10B-C). D: single optical section (0.7 μm) from this stack at z = 1.5 μm showing the non-columnar olfactory neuropil C. It is not possible to decide if neuropil C does in fact connect with the bases of the glomeruli. E: Single optical section (0.44 μm) from another specimen. The olfactory glomeruli are subdivided into a cap region (Cp) that shows only synapsin immunoreactivity (red) and a subcap (Sc) and base (Ba) region in which synapsin immunoreactivity overlaps with RFamide-like immunoreactivity (yellow/orange color). The boxed area is shown in a higher magnification in Fig. 11.

As noted above, the ON is composed of numerous glomeruli that display strong SYNir and are arranged parallel to each other around the periphery of the lobe (Fig. 10, 11). Examining single optical sections from a z stack gives an idea of the dense packing of these glomeruli (Fig. 10A1, A2). They are elongate, cylindrical structures and their distal part is slightly larger than the proximal part. The glomeruli have a length of around 150 μm. A 3D reconstruction (Fig. 10B) confirmed that, as seen in tangential sections (Fig. 3A, 11A), the cross-sectional profile of these structures is more or less round. The diameter of the glomeruli in cross sections is around 20 μm. The periphery of the ONs is entirely packed with glomeruli with the exception of two spared spaces, the foramina, where fiber bundles enter or exit the lobes. These are the median foramen (mF; Fig. 3E, F, 4D, 8, 9A, C, 11B), through which the neurites from cluster (9) interneurons pass into the lobe and through which the olfactory globular tracts exits is, and the posterior foramen (pF), through which the axons of cluster (10) neurons enter the lobe (Fig. 3C, D, 4C; see also Fig. 1B). Double labeling for SYNir and RFir revealed a regionalization of the glomeruli. Whereas SYNir is present throughout the entire glomeruli, the cap region is devoid of RFir (terminology according to [59]). However, RFir is present in the base region and is particularly strong in the subcap region (Fig. 11B, C1, C2, D, E).

**Figure 12:**
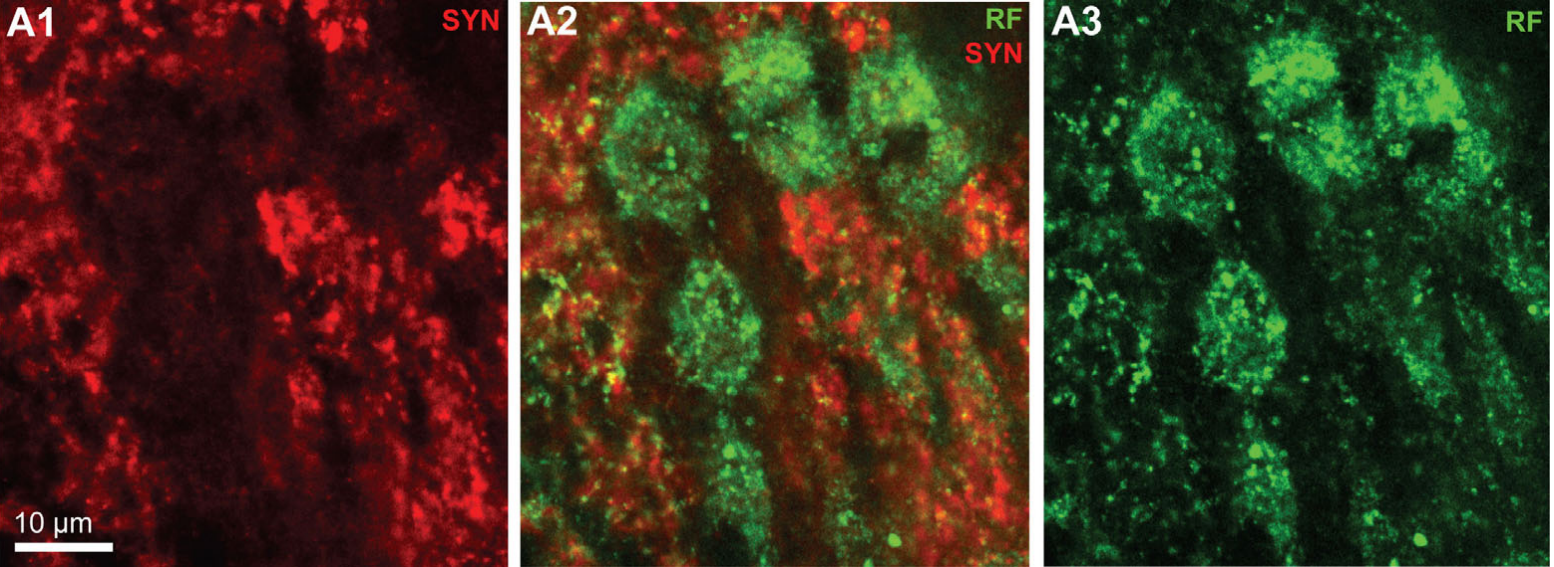
**Higher magnification of the non-columnar olfactory neuropil (merged patches C/D) shown in Fig. 10E.** Double labeling for synapsin immunoreactivity (SYN; red) and RFamide-like immunoreactivity (RF; green); confocal laser scan microscopy, single optical section (0.44 μm). Numerous round RFamide-like immunoreactive swellings of ca 10 μm diameter, putative non-synaptic peptide release sites, are embedded within the synapsin immunoreactive neuropil, but both labels are not co-localized.

Apart from the glomeruli, a second type of neuropil in the ONs, the non-columnar olfactory neuropil (ncON), displays strong SYNir, 5HTir and RFir. Six patches of this diffusely structured neuropil can be reproducibly identified in different specimens and will be denoted with capital letters A-F in the following (see summary diagram Fig. 1B). Patch A of the ncON (Fig. 3B, 4B, 11A) is seen in ventrally located tangential sections of the ON in a medio-posterior position to the main ON. It stretches further dorsally towards the point where the extra olfactory lobe joins the main lobe (Fig. 4B). Patch B is located anteriorly to the median foramen (Fig. 3G, 8, 9A, C). In sections dorsal to the median foramen, it merges with patch F that is located posterior to this foramen. The merged patches B and F thus line the dorsal part of the median foramen (Fig. 3H, 5B2, 8, 9A, C, 10A1, A2, 11, 13A). Hence, it appears that the thick bundles of cluster (9) interneurons on passing into the olfactory lobe are surrounded by ncON. Patch C of the ncON is closely linked to patch D by a broad neuropil bridge. Both patches are located antero-laterally in the ONs and are flanked by the columnar neuropil (Fig. 3C–H, 4C, D, 7B2, 9C, 10A1, A2, C, 11B–E). Patch E of the ncON is associated with the posterior foramen from where it stretches further dorsally, there being embedded within columnar neuropil (Fig. 3D, E, 4D, 11B, C1, C1, 9C). Patch E is linked to patch F by a neuropil bridge at the level of those sections that show the median foramen (Fig. 9F). Patches C-E are located very close to the proximal bases of olfactory glomeruli and in some places even seem to merge with the glomeruli (Fig. 10A1, 11B–C). However, analyses of single optical sections (Fig. 10C, 11D) could not unequivocally answer the question if fiber connections between the ncON and the glomeruli exist. Double labeling of the ncON showed numerous round RFir swellings to be embedded within the SYNir neuropil (Fig. 11B–E). However, close inspection of single confocal sections failed to show a co-localization of both labels (Fig. 12) suggesting that, if RFamide-like peptides are released into the ncON, synapsins are not associated with these release sites (at least as far as detectable with our methods).

### Deutocerebrum: other neuropils

The median antenna 1 neuropil (MAN) extends across the brain posterior to the protocerebrum behind the cerebral artery and is on both sides flanked by the arms of the olfactory globular tract (Fig. 6D1, D2, 7A1, A2, 8). Anteriorly, it seems to be continuous with the protocerebral neuropils. It displays both, strong SYNir and Rfir, the latter being distributed in rather coarse profiles (Fig. 6D2, 8). The lateral antenna 1 neuropil (LAN) in decapod crustaceans is known to receive afferents from the mechanoreceptors of the antenna 1 (Sandeman et al. 1992, 1993). In *C. clypeatus*, it caudally adjoins the median antenna 1 neuropil (Fig. 3F–H, 4D–F, 5A, B, 6A, 7A1, 7, 9A–C, 13A) and in some sections seems to be connected to this neuropil (Fig. 3F, 4F, 5A, 7A2). Similar to the non-columnar olfactory neuropil, large, round RFir profiles are embedded in the SYNir neuropil of this structure (Fig. 8).

**Figure 13:**
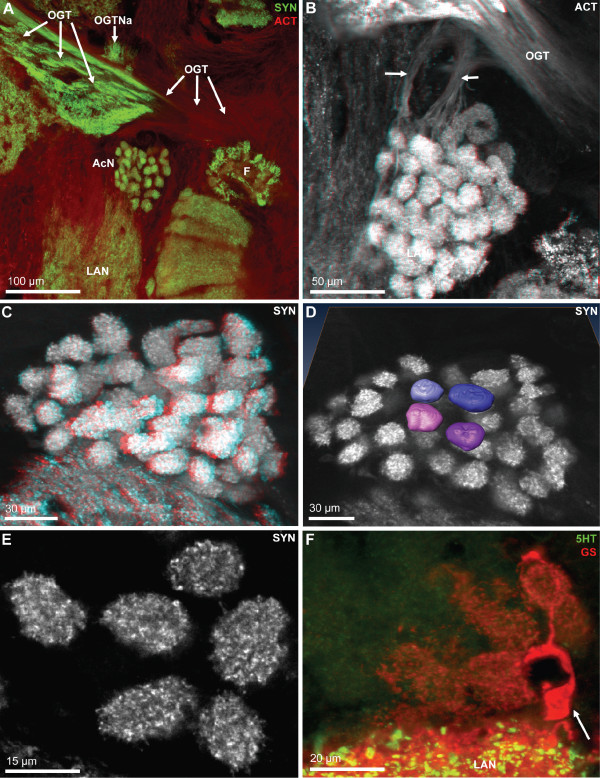
**Details of the accessory lobe (AcN); confocal laser scan microscopy.** A: Double labeling for synapsin immunoreactivity (SYN; green) and actin (ACT; red). The accessory lobe is located close to the exit point of the olfactory globular tract (OGT) from the olfactory lobe. Other abbreviations: F patch F of the non-columnar olfactory neuropil, LAN lateral antenna 1 neuropil, OGTNa accessory olfactory globular tract neuropil. B: Histochemical localization of actin (ACT) shows two fiber bundles (arrows) that extend from the accessory lobe towards the olfactory globular tract (OGT) but seem to pass underneath it. Color coded three dimensional visualization of a confocal stack, use red-green glasses to view. C: Color coded three dimensional visualization of a confocal stack (use red-green glasses to view) of a synapsin labeled specimen shows that the accessory lobes is composed of an array of evenly spaced spherical glomeruli. D: a surface reconstruction of four glomeruli obtained from a z-series of confocal images (synapsin immunoreactivity) that were directly loaded into Amira and processed for semiautomatic segmentation using Amira's "wrap" module. E: single optical section of the glomeruli (synapsin labeling) to show the regular structure of the synaptic neuropil within the glomeruli. F: Double labeling for serotonin immunoreactivity (5HT; green) and glutamine synthetase-like immunoreactivity (GS; red). A single glutamine synthetase positive cell (arrow) just adjacent to the lateral antenna 1 neuropil (LAN) extends branches towards and penetrates into several glomeruli of the accessory lobe.

The accessory neuropil (AcN) or accessory lobe is another conspicuous feature of the *C. clypeatus *deutocerebrum. It is located medially to the ON, close to where the olfactory globular tract exits the latter (Fig. 3G, H, 4E, F, 6D1, D2, 7, 8, 13A). The accessory lobe is composed of about 50–80 small, spherical glomeruli, all of which have a diameter of around 10–15 μm (Fig. 13B–D). In specimens processed for SYNir, the synaptic areas of the glomeruli appear well separated from each other and the glomeruli do not show any recognizable substructures (Fig. 13C–E). Actin labeling reveals a bundle of fibers extending anteriorly from the accessory lobe towards the olfactory globular tract (Fig. 13B). However, we were not able to determine if this fiber bundle connects to the latter tract. Fig. 13F shows a specimen processed for GSir. The arrow identifies the soma of a glia cell, the processes of which branch and associat with several glomeruli in the accessory lobe.

### Tritocerebrum

**Figure 14:**
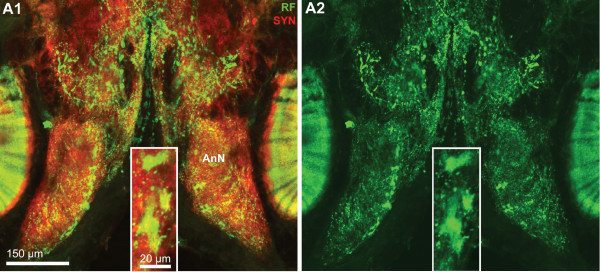
**Details of the antenna 2 neuropil (AnN).** Double labeling for synapsin immunoreactivity (SYN; red, A1) and RFamide-like immunoreactivity (RF; green, A1, A2). Note that in addition to numerous small peptidergic profiles which are embedded within the synaptic neuropil, several large peptidergic profiles are present that may represent non-synaptic release sites (inset).

The antenna 2 neuropil (AnN) receives afferent input from the second antenna but also provides motor innervation for the muscles that move this antenna [52]. It is located posteriorly to the lateral antenna 1 neuropil and is visible in the middle of the section stacks (Fig. 4E, F, 5D, 6B1, B2, 7A, 10C, 14). With neither SYNir nor RFir could we recognize any compartmentalization or substructures in this neuropil (Fig. 14). Yet, once again we observed large and very prominent RFir swellings in the neuropil suggesting the presence of peptide release sites (Fig. 14 inset).

### Eyestalk neuropils: the lateral protocerebrum – medulla terminalis and hemiellipsoid body

**Figure 15:**
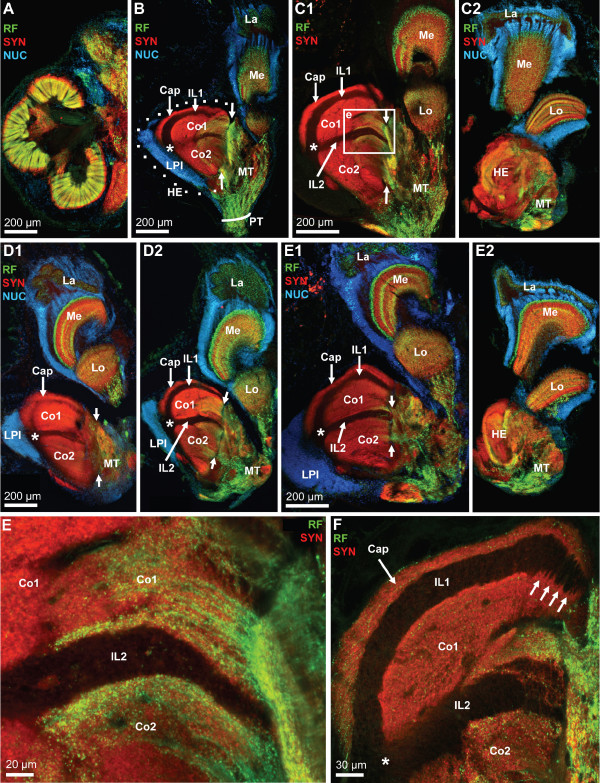
**The eyestalk neuropils as shown in vibratome sections that were double labelled (E, F) for synapsin immunoreactivity (SYN; red) and RFamide-like immunoreactivity (RF; green), or triple labeling for these two substances plus the nuclear marker (NUC; A-E2); conventional fluorescence combined with the Apotome structured illumination technique for optical sectioning (A-E2) and confocal laser scan microscopy (E, F).** A, B and D are rendered at the same scale to compare the size of a median hemi brain (A; see also Fig. 3D) with that of the eyestalk neuropils. The eyestalks contain the lateral protocerebrum which is composed of the medulla terminalis (MT) and the hemiellipsoid body (HE). Furthermore, the four optic neuropils lamina (La), medulla (Me), lobula (Lo) and lobula plate are enclosed in the eyestalks. The low power views presented in B-E2 show four different eyestalks to show the individual variation between specimens. The hemiellipsoid body (HE; encircled by a dotted line in B) is a large spherical neuropil that is associated with a compact, laterally situated cluster of densely packed neurons, the lateral protocerebral interneurons (LPI). With the synapsin label, several neuropil compartments are visible within the hemiellipsoid body, the peripheral cap neuropil (Cap) which is separated by the unlabelled intermediate layer 1 (IL 1) from the strongly synapsin immunoreactive core neuropil 1 (Co1). A second unlabeled intermediate layer (IL 2) separates core neuropil 1 from the more proximally located core neuropil 2 (Co2). Asterisks in B-E2 and F mark the point where the two intermediate layers meet. The boxed area in C1 is shown at a higher magnification in E. The demarcation between the medulla terminalis (MT) and the hemiellipsoid body is difficult to draw. The opposed arrows in B-E tentatively mark the border between these two structures. From the medulla terminalis, strongly labeled RFamide-like immunoreactive fibers invade the core neuropils 1 and 2 (Co1, Co2) of the hemielliposid body where they terminate in a circumscribed field with very fine varicosities (E, F). Intermediate layers IL1 and IL2 are unlabeled. The arrows in F identify patches of neuropil that seem to extend across the intermediate layer 1.

Apart from the medial portion of the brain in the head capsule, another substantial part of the brain is located within the eyestalks. Fig. 15A, B and 15D feature images of the median brain and the eyestalk neuropils reproduced at the same scale to make the point that specifically one of the eyestalk neuropils, the hemiellipsoid body, almost matches the size of the olfactory lobes (ON). The medial protocerebrum is connected to the eyestalk neuropils via the protocerebral tract (PT; Fig. 15B), which includes the olfactory globular tract that ascends from the deutocerebrum. The eyestalks contain the lateral protocerebrum which is composed of the medulla terminalis (MT) and the hemiellipsoid body (HE). Furthermore, the four optic neuropils lamina (La), medulla (Me), lobula (Lo) and a small proximal lobula neuropil (LoP) are enclosed in the eyestalks (Fig. 15A–D, 18A). Because the spatial arrangement of the eyestalk neuropils is rather complicated, Fig. 15 presents low power views of four different specimens to demonstrate these relationships and also to provide an idea of the level of structural variation between individuals.

**Figure 16:**
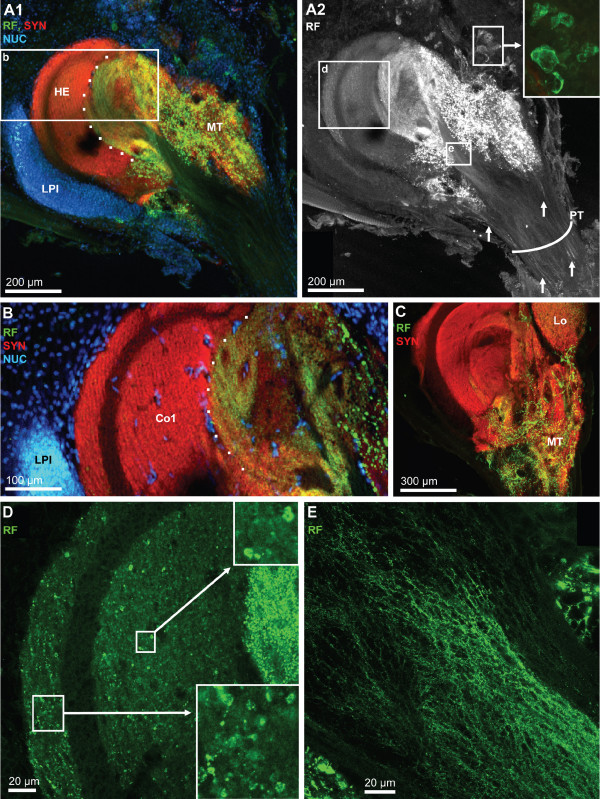
**Details of the hemielliposid body and the medulla terminalis; triple labeling for synapsin immunoreactivity (SYN; red), RFamide-like immunoreactivity (RF; green), plus the nuclear marker (NUC) shown in conventional fluorescence combined with the Apotome structured illumination technique (A1, B) and confocal laser scan microscopy (A2, C-E).** A1: a massif cluster of lateral protocerebral interneurons (LPI) is associated with the hemiellipsoid body (HE). The dotted line demarks the field of strong innervation with peptidergic neurites. The medulla terminalis (MT) is filled with a loose meshwork of peptidergic fibers. The boxed area is shown at a higher magnification in B. A2: same specimen as in A1 but visualized with confocal microscopy and showing only the RFamide channel. Arrows identify single, peptidergic fibers within the protocerebral tract (PT). Peptidergic interneurons are associated with the medulla terminalis (inset). The boxed areas d and e are shown at a higher magnification in D and E. B: Already at moderate magnification, the core neuropil (Co1) displays a layered appearance. The dotted line demarks the field of strong innervation with peptidergic neurites. The cluster of lateral protocerebral interneurons is also visible (LPI) as are single cell nuclei (presumably endothelial cells of blood vessels) within the core neuropil. C: The medulla terminalis (MT) is filled with a loose meshwork of peptidergic fibers. D: Higher magnification of the boxed area in A2. Both the cap and core 1 neuropils are filled with numerous very small RFir profiles some of which are arranged in a string (see insets) suggesting an intense peptidergic innervation of these neuropils. E: higher magnification of the boxed area in A2 showing a meshwork of very fine peptidergic fibers within the olfactory globular tract close to its entrance into the medulla terminalis/hemiellipsoid body complex.

This paragraph will focus on the neuropils that constitute the lateral protocerebrum, the closely associated hemiellipsoid body (enclosed by dots in Fig. 15B) and the medulla terminalis (MT). The lobula ontogenetically derives from the medulla terminalis and hence is part of the lateral protocerebrum [60]. Nevertheless, in the present account it will be described together with the other two visual neuropils. The demarcation between the medulla terminalis and the hemiellipsoid body is difficult to draw and we will here use the RFir as a somewhat arbitrary landmark. The opposed arrows in Fig. 15B–E mark the border between the medulla terminalis which is filled by a loose network of peptidergic fibers and the hemiellipsoid body that displays strong SYNir. A cluster of RFir cell bodies flanks the medulla terminalis laterally and probably gives rise to at least some of the RFir innervation of this neuropil (inset in Fig. 16A2). In low power views, strongly RFir fibers in the protocerebral tract (Fig. 15B) seem to spread out and give rise to an intense peptidergic innervation of the medulla terminalis. At a higher magnification, the protocerebral tract carries only few distinct RFir fibers (Fig. 6D1, arrows in Fig. 16A2). Yet, even higher magnifications of the protocerebral tract close to the medulla terminalis (see frame in Fig. 16A2) reveals an extensive network of very fine RFir fibers within this tract (Fig. 16E). We could not determine if this network represents efferent fibers from the median brain to the lateral protocerebrum or if they originate from the local RFir neurons associated with the medulla terminalis.

**Figure 17:**
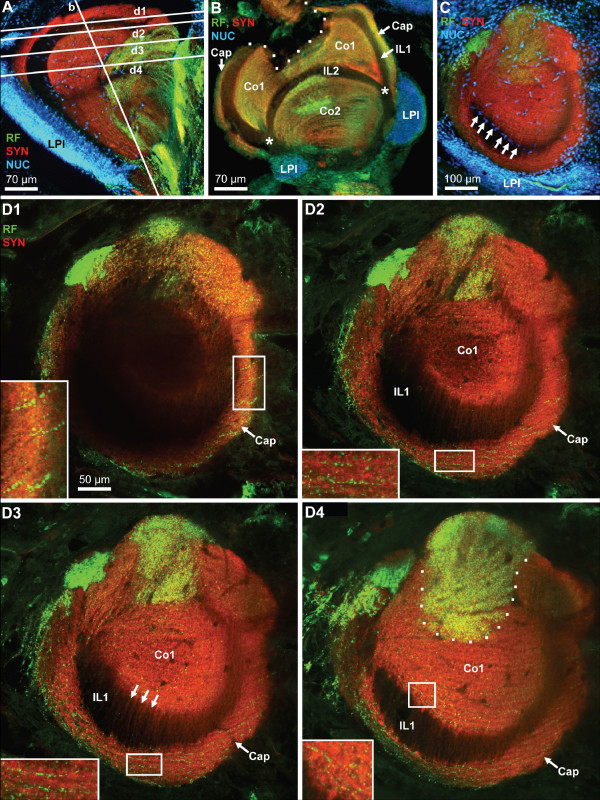
**High power views of the hemiellipsoid body to reveal the lamellar organization of the cap and core neuropils.** Triple labeling for synapsin immunoreactivity (SYN; red), RFamide-like immunoreactivity (RF; green), plus the nuclear marker (NUC) shown in conventional fluorescence combined with the Apotome structured illumination technique (A-C) and confocal laser scan microscopy (D1–D4). A: the position of sections D and D1–D4 is indicated here. The cluster of lateral protocerebral interneurons (LPI) is identified. B: transverse section of the hemiellipsoid body demonstrating the arrangement of the cap (Cap), core 1 (Co1) and core 2 (Co2) neuropils as well as the intermediate layers 1 and 2 (IL1, IL2; the dotted lines delineates a damaged region of the tissue). The extensive cluster of lateral protocerebral interneurons (LPI) is seen to stretch around both sides of the hemiellipsoid body. C: A tangential section with the nuclear counter stain reveals the presence of cell nuclei that are interspersed in the core 1 neuropil, presumably belonging to endothelial cells of blood vessels. In addition, the interface between intermediate layer 1 and core neuropil 1 is lined with cell nuclei (arrows). The extensive cluster of lateral protocerebral interneurons (LPI) stretches around the hemiellipsoid body. D1–D4: a series of tangential optical sections through the hemiellipsoid body (the positions are indicated in A). Note the lamellar organization of the cap and core neuropil. The entire image stack is composed of 29 optical sections of 0.76 μm thickness covering z = 21.2 μm. The four single images are projections of 3 optical sections covering z = 0 – 1.5 μm (D1; the most superficial section), z = 3.8 – 5.3 μm (D2), z = 9.1 – 10.6 μm (D3), and z = 18.2 – 19.7 μm (D4). The Cap (Cap) and core 1 (Co1) neuropils are visible as well as the intermediate layer 1 (IL1). The insets in sections D1–D3 demonstrate the string-like arrangement of tiny RFir profiles in the cap neuropil which are arranged parallel to the lamellae, and the inset in D4 shows similar profiles in the core neuropil 1. In sections D3 and D4, strands of lightly synapsin immunoreactive material seem to span across the intermediate layer 1 (arrows in D3). The dotted line in D4 identifies that sector of the core 1 neuropil that is strongly invaded by RFir fibers.

The hemiellipsoid body is a large (ca. 300 μm in diameter), spherical neuropil that is associated with a compact, laterally situated cluster of densely packed neurons, the lateral protocerebral interneurons (LPI; Fig. 15A–E, 16A1, B, 17A–C). With the synapsin label, several neuropil compartments are visible within the hemiellipsoid body. The peripheral cap neuropil is separated by the unlabelled intermediate layer 1 (IL 1) from the strongly SYNir core neuropil 1 (Co1). A second unlabeled intermediate layer (IL 2) separates core neuropil 1 from the more proximally located core neuropil 2 (Co2; Figs. 15, 16, 17). We suggest that the unlabelled intermediate layers 1 and 2, which have a common origin (asterisks in Fig. 15B, C1, D, E1, F, 17B), may be the sites where the fibers of the lateral protocerebral interneurons penetrate into the hemiellipsoid body. From the medulla terminalis, strongly labeled RFir fibers invade the core neuropils 1 and 2 of the hemielliposid body, where they terminate in a circumscribed field with very fine varicosities (Fig. 15E). This field is labeled with a dotted line in figures 16A1, B, 17D4). Apart from this strongly labeled field, high power views of the cap and those regions of the core neuropils that in low power views did not seem to display RFir, nevertheless demonstrate a weak but distinct peptidergic innervation. Fig. 16D demonstrates that both the cap and core 1 neuropils are filled with numerous very small RFir profiles, some of which are arranged in a string, suggesting an intense peptidergic innervation of these neuropils. This string-like appearance is even more apparent in tangential sections (Fig. 17; see below). Already at low magnification, in preparations with SYNir, the core neuropils one and two have a layered appearance (Fig. 15E1, 16A1). Higher power views reveal that not only the core but also the cap neuropils are clearly organized into parallel layers, or lamellae (Fig. 16B, 17D2–D4). Fig. 17B shows a transverse section of the hemiellipsoid body, the position of which is indicated in Fig. 17A. This section nicely demonstrates the arrangement of the intermediate layers 1 and 2 (the dotted lines delineates a damaged region of the tissue). Furthermore, the extensive cluster of lateral protocerebral interneurons is seen to stretch around both sides of the hemiellipsoid body. Fig. 17D1–D4 shows a series of tangential optical sections through the hemiellipsoid body, the positions of which are indicated in Fig. 17A. Section D1 is the most superficial and shows the cap region as well as the intermediate layer 1. Section D2 is slightly deeper and touches the upper part of core neuropil 1. The insets in sections D1–D3 demonstrate the string-like arrangement of tiny RFir profiles in the cap neuropil, and the inset in D4 shows similar profiles in the core neuropil 1. In sections D3 and D4 strands of lightly SYNir material (arrows in D3) seem to span across the intermediate layer 1 thus connecting the core neuropil 1 and the cap neuropil (see also arrows in Fig. 15F). This hemiellipsoid body sector, which is strongly invaded by RFir fibers, is identified by a dotted line in section D4. Sections with the nuclear counter stain reveal the presence of cell nuclei that are interspersed in the core 1 neuropil (Fig. 16B, 17C). These nuclei presumably belong to endothelial cells of blood vessels. In addition the interface between intermediate layer 1 and core neuropil 1 is lined with cell nuclei (arrows in Fig. 17C).

### The eyestalk neuropils: lamina, medulla, lobula (optic neuropils)

**Figure 18:**
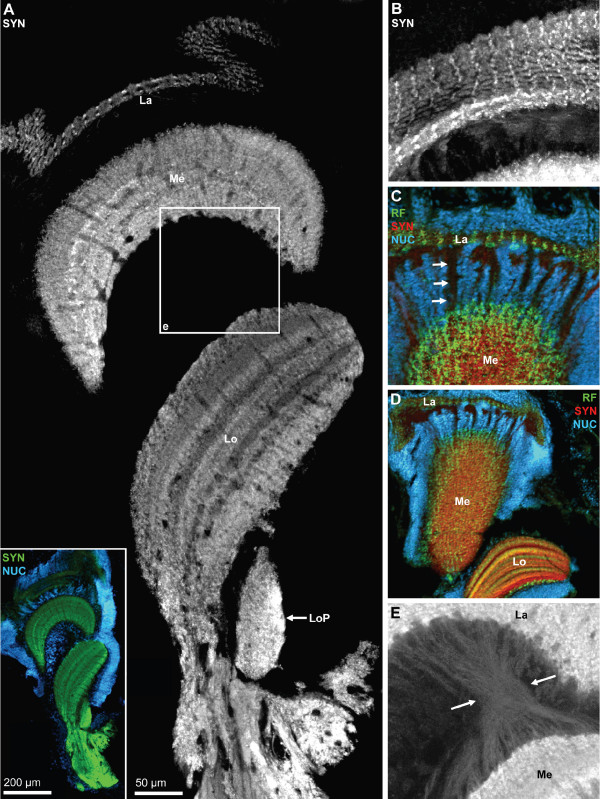
**The optic neuropils.** A: overview (photomontage, confocal laser scan microscopy), vibratome section showing synapsin immunoreactivity (SYN; green in the inset), plus the nuclear marker (NUC; blue in the inset only; Apotome structured illumination technique). The three retinotopic neuropils from distal to proximal are the lamina (La), medulla (Me), Lobula (Lo) which is associated with an additional proximal lobula neuropil (LoP). The inset shows that the optic neuropils are surrounded by a cortex of neuronal somata. The boxed area is shown in E in a higher magnification. B: same specimen as A; maximum projection of several confocal sections to show the plexiform layer of the lamina. C, D: Triple labeling for synapsin immunoreactivity (SYN; red), RFamide-like immunoreactivity (RF; green), plus the nuclear marker (NUC) shown in conventional fluorescence combined with the Apotome structured illumination technique. In these tangential sections of the medulla, a regular arrangement of the labeled profiles signifies the ordered, retinotopic organization of this neuropil. E: higher magnification of the boxed area in A. In this image, contrast and brightness levels were artificially elevated, so that unspecific background staining reveals the presence of the inner optic chiasm, a cross-over of the fibers that connect the medulla and the lobula (enclosed between the arrows).

**Figure 19:**
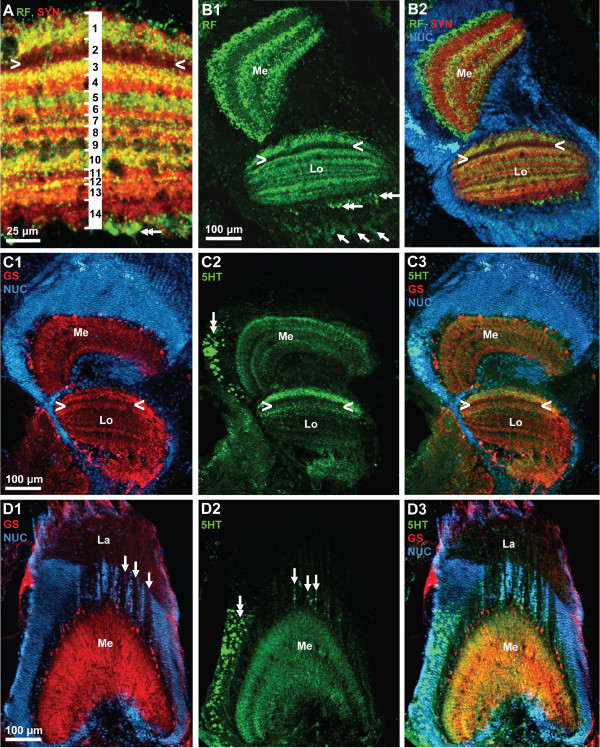
**A, B: lobula (see A) and medulla (Me) and lobula (Lo; B1, B2); triple labeling for synapsin immunoreactivity (SYN; red), RFamide-like immunoreactivity (RF; green), plus the nuclear marker (NUC) shown in conventional fluorescence combined with the Apotome structured illumination technique.** In the medulla, RFir is localized in three distinct parallel layers. In the lobula (see A), fourteen different layers can be recognized with this technique. Arrowheads label a conspicuous weakly labelled layer. Double arrows identify proximally located, large RFir profiles associated with the lobula. Arrows identify RFir somata of visual interneuons. C1–C3: lobula (Lo) and medulla (Me); triple labeling for serotonin immunoreactivity (5HT; green), glutamine synthetase-like immunoreactivity (RF; red), plus the nuclear marker (NUC; blue) shown in conventional fluorescence combined with the Apotome structured illumination technique. Arrowheads label a conspicuous weakly labelled layer (compare B2). D1–D3: lamina (Lo) and medulla (Me); triple labeling for serotonin immunoreactivity (5HT; green), glutamine synthetase-like immunoreactivity (RF; red), plus the nuclear marker (NUC; blue) shown in conventional fluorescence combined with the Apotome structured illumination technique. Arrows in D1 label the course of fiber bundles that link the lamina and the medulla. Arrows in D2 identify serotonergic somata located between the lamina and the medulla. Double arrows in D2 label serotonergic neurons associated laterally with the medulla.

In decapod crustaceans, the visual input from the compound eyes is processed in three columnar optic neuropils, the lamina (lamina ganglionaris according to the older terminology), the medulla (medulla externa) and the lobula (medulla interna) all of which are enclosed in the eyestalk (compare Fig. 15, 18). Recent studies provide evidence for a fourth neuropil in crabs associated with the lobula, the lobula plate [61]. All neuropils can be identified by SYNir in *C. clypeatus *(Fig. 18A) but without any additional markers that would allow the visualization of the fiber composition it is not possible at the moment to decide if the additional proximal lobula neuropil in *C. clypeatus *corresponds to the lobula plate of brachyuran crabs. As in Drosophila, the labeling in the lamina is much weaker with the SYNORF antibody than in the other neuropils (Fig. 18A, B). SYNir identifies the plexiform layer of the lamina. In the projection of a confocal image stack the geometrical layout of the lamina is visible reflecting the arrangement of optic cartridges (Fig. 18B). Furthermore, a regular pattern of small RFir profiles is present in the plexiform layer of the lamina (Fig. 18C). The optic neuropils are surrounded by a layer of visual interneurons that we did not chart in any detail (inset in Fig. 18A). Many cell bodies are also present between the lamina and the medulla (Fig. 18C, D, 19), and the medulla and lobula (Fig. 19). The arrangement of cell nuclei between the lamina and the medulla, in a kind of negative image, reveals the course of fiber bundles that connect the lamina and the medulla (arrows in Fig. 18C, 19D1, D2).

In synapsin labeled preparations, it becomes clear that the medulla and lobula are composed of several parallel layers (Fig. 15, 18a). Darker, irregularly arranged areas in the medulla and lobula neuropil presumable show the course of blood vessels (Fig. 18a). A small but distinct additional neuropil is proximally associated with the lobula (LoP; Fig. 18A and inset). In tangential sections of medulla labelled for SYNir and RFir, a regular arrangement of the labeled profiles signifies the ordered, retinotopic organization of the medulla (Fig. 18D). In cross sections of the medulla, RFir is clearly localized in three distinct parallel layers (Fig. 19B1, B2). This neuropil is also strongly innervated by a cluster of serotonergic visual neurons located at the side of it (double arrow in Fig. 19D2). Additionally, serotonergic somata are located in the cell group between the lamina and the medulla (arrows in Fig. 19D2). Within the medulla neuropil, 5HTir is also arranged in parallel layers although less distinct than RFir (Fig. 19C2, C3). Cell somata with strong GSir, presumably ensheathing glia cells, surround the medulla laterally and distally and give rise to a strong glutamine synthetase signal within the neuropil (Fig. 19C1, D1). In an image with SYNir, in which the contrast and brightness levels were artificially elevated, unspecific background staining reveals the presence of the inner optic chiasm, a cross-over of the fibers that connect the medulla and the lobula (enclosed between the arrows in Fig. 18E). GSir is also strong in the third optic neuropil, the lobula. Within the neuropil, GSir shows the layered appearance of the lobula (Fig. 19C1, C3) that is also apparent with RFir (Fig. 19A, B1, B2) and 5HTir (Fig. 19C2). At least fourteen layers could be identified with RFir and SYNir but we did not analyze the layering in more detail (Fig. 19A). With all three markers, one conspicuous layer in the lobula is devoid of labeling (arrowheads in Fig. 19A, B1, B2, C1–3). A population of weakly labelled RFir cell somata is associated proximally with the lobula (single arrows in Fig. 19B1). Strongly RFir profiles line the most proximal neuropil layer of the lobula (double arrows in Fig. 19A, B1).

## Discussion

The terrestrial hermit crab *C. clypeatus *has evolved a sense of aerial olfaction. Previous behavioral studies have provided evidence that these animals are very effective in detecting food from a distance and in responding to airborne odors. Here we confirm that these behavioral observations are paralleled by a significant elaboration of brain areas taking part in olfactory processing, as has already been noted by Beltz and co-workers [42] who reported that *C. clypeatus *has a fairly high number of elongate olfactory glomeruli compared to other Crustacea (see below). We show that the primary olfactory centers (olfactory lobes) in this species dominate the brain and are equipped with a side olfactory lobe and that the secondary olfactory centers (hemiellipsoid bodies) are also very large. The hemiellipsoid bodies which receive a massif input of olfactory projection neurons are organized into parallel neuropil lamellae. Furthermore, our data suggest that the organization of the visual centers and those areas associated with antenna two suggest that the visual and mechanosensory skills of *C. clypeatus *are similar to those of their marine relatives.

### The central olfactory pathway – deutocerebral neuropils

In malacostracan crustaceans, afferent chemosensory input from the olfactory receptor neurons housed in the aesthetascs on the paired first antennae is processed in conspicuous deutocerebral neuropil centers, the bilaterally arranged olfactory lobes. These consist of cone-like areas of dense synaptic neuropil, the glomeruli, which are arranged around the periphery of the lobe with the apices pointing to the centre of the lobe (overviews in [52,57,62-66]). Mechanosensory and non-olfactory chemosensory input from the first antennae is processed in the lateral antenna 1 neuropil (LAN) and the medial antenna 1 neuropil (MAN; [65,67,68]). Schachtner and coworkers [43] have summarized cellular characteristics of the various classes of interneurons that are associated with the glomeruli of the olfactory lobes. A longitudinal subdivision of the glomeruli into the cap, subcap, and base regions has been well documented in crayfish, clawed and clawless lobsters [59,62,66,69-71] and the olfactory glomeruli of *C. clypeatus *conform to this design. The known numbers of glomeruli varies considerably across the Reptantia ranging from ca. 200 in crayfish to more than 1000 in spiny lobsters [43]. It has been speculated that there is a relationship between the number of glomeruli and the classes of different olfactory receptor neurons on the antennae and hence the number of different odors that the animals can resolve (discussed in [42,43]). Recently, Mellon suggested [25] that "the number of glomeruli in the olfactory lobe should provide a numerical value close to, if not identical with, the actual number of expressed odorant receptors across the olfactory receptor neuron array". Beltz and co-workers [42] counted the numbers of olfactory glomeruli in 17 species of reptantian crustaceans and attempted to correlate these numbers to life styles, habitat, phylogenetic affinities, and numbers of olfactory sensilla. Although their study did not reveal a clear-cut correlation of glomerular numbers with any of these factors but instead suggested that problems of size, sensitivity and selectivity have all interacted during evolution of crustacean olfactory systems, a closer look at these author's data nevertheless seems warranted. Glomerular numbers were highest – between 960 and 1330 – in three species of Achelata, clawless lobsters with a large body size. Among the remaining 14 representatives of Homarida, Astacida, Thalassinida, Anomura, and Brachyura, *Coenobita clypeatus *had the highest number of glomeruli (ca. 800) and ranked third concerning olfactory lobe volume and glomerular volume [42] indicating the presence of a quite sophisticated olfactory system in this organism.

The neurochemistry of the olfactory interneurons that synapse within the glomeruli is complex (review [43]). The variety of neuroactive substances that has been localized in the olfactory system include histamine, serotonin and GABA [54,70,72,73], and the neuropeptides Substance P, FMRFamide, small cardioactive peptide_b _[51,59,70,74-77] as well as a tachykinin-related peptide [78], SIFamide [79] and a novel member of the allatostatin family [80]. Despite this detailed knowledge about the cellular composition of the glomeruli and although numerical aspects concerning the glomeruli were analyzed in a comparative approach [42], comparative studies of the anatomy of the olfactory lobe and the shape of its glomeruli are not available for the Malacostraca. Table 1 provides a list of histological studies in which the shape of the olfactory glomeruli in various malacostracan crustaceans is visible. In most Malacostraca examined so far, the olfactory glomeruli are roughly conical or cylindrical columns (but compare to Stomatopoda which seem to have spherical glomeruli; see [81]) arranged radially around the periphery of the lobes. Yet, the shape and size of these glomeruli display a considerable variability. The tall, narrow, and elongate shape of the glomeruli reported here for *C. clypeatus *seem to represent one extreme end of this variability. Of particular importance for this comparison are of course members of the closest related taxa, the Thalassinida and Brachyura. In a synapsin labeled specimen of *Callianassa australiensis *(Thalassinida) the glomeruli are shaped like a barrel [82] and the same seems to be the case in brachyuran crabs ([47,51,52], and Fig. 2B in [43]). Among the few anomuran crabs analyzed so far are squat lobsters of the genus *Munida *[41,83], which as members of the Galatheidae are not closely related to the Coenobitidae (Fig. 1). In *Munida sarsi*, the olfactory glomeruli are conical and are only twice as long as they are wide (Fig. 2B in [83]). Furthermore, one image of the olfactory neuropil in *Pagurus bernhardus *(Paguridae) is available (Fig. 2C in [43]).

**Table 1:** studies on the olfactory lobes in various malacostracan crustaceans.

**Eumalacostraca**

**Stomatopoda**
*Neogonodactylus oerstedii *[81]

**Euphasiacea**
*Meganyctiphanes norvegica *[137]

**Peracarida**
*Neomysis integer *[137,138]
*Leptomysis lingvura *[117]
*Hemimysis margalefi *[117]
*Lophogaster typicus *[117]

**Decapoda, Pleocyemata**
**Caridea**
*Macrobrachium rosenbergii *[83]
*Palaemonetes pugio *[82]

**Reptantia**
**Achelata**
*Panulirus argus *[52,59,63,71]
*Jasus novaehollandiae *[40]
*Ibacus peronii *[40]

**Homarida**
*Homarus americanus *[40,70,133,139]

**Astacida**
Various crayfish species [47,52,58,62-64,69,78]

**Thalassinida**
*Callianassa australiensis *(syn.*Trypaea australensis; *[40,82])

**Anomura**
*Munida quadrispina *[41]
*Munida sarsi *[83]

**Brachyura**
*Scylla serrata *[52]
*Hyas araneus *(larvae; [51])
*Hemigrapsus sanguineus *[47]

a list of studies that provide histological images of the olfactory lobes and the shape of the olfactory glomeruli in various malacostracan crustaceans. Only contemporary literature is considered in which histological photomicrographs of the olfactory lobes are shown thereby excluding e.g. the reports [25,46] in which mostly schematic drawings are provided. The phylogenetic terminology is according to [44].

In order to broaden the taxonomic horizon for a comparative analysis of anomuran olfactory systems we set out to analyze the olfactory neuropils in some additional anomuran taxa of the subgroup Paguroidea which are closely related to the Coenobitidae (Harzsch and Hansson, unpublished data): *Clibanarius erythropus, Diogenes pugilator*, and *Calcinus elegans *as members of the Diogenidae (compare Fig. 1) and *Pagurus bernhardus *again, as a member of the Paguridae. Furthermore, we have analyzed the giant robber crab *Birgus latro *[84] which as a member of the Coenobitidae is most closely related to *Coenobita clypeatus*. From this preliminary study it would appear that the elongate shape of the glomeruli in *C. clypeatus *(at least five times as long as theay are wide) and also in *B. latro *marks one end of the range, whereas *D. pugilator *with glomeruli that are only twice as long as they are wide marks the other end. *C. erythropus, C. elegans*, and *P. bernhardus *fall in between these two extremes (Harzsch and Hansson, unpublished data). As mentioned above, *C. clypeatus *has a relatively high number of glomeruli (ca. 800) compared to other Decapoda [42]. The need to pack many glomeruli in a radial array and a restricted amount of space may promote the evolution of these elongate glomeruli. A comparison with the marine hermit crabs that we analyzed and with the studies listed in table 1 also reveals that the existence of an additional side olfactory lobe as shown here for *C. clypeatus *is not a typical feature of other malacostracan crustaceans. However, one of its nearest relatives, *B. latro*, has an olfactory neuropil that is even composed of three sublobes [84].

Clearly, these anatomical features in concert with the high number of olfactory glomeruli [42] and the remarkable neuroarchitecture of their secondary olfactory processing areas (see next section) suggest the central olfactory system of *C. clypeatus *and also of *B. latro *[20,84] to be well adapted for aerial olfaction. These neuroanatomical findings can explain those behavioral reports that have provided evidence that the Coenobitidae are very effective in responding to volatile odors and possess an excellent sense of distance olfaction [20-22].

### The central olfactory pathway – lateral protocerebrum

The lateral protocerebrum (medulla terminalis, glomeruli centrales, and hemiellipsoid body; [63]) receives a massive input from the olfactory globular tract that originates from the cluster (10) of projection neurons associated with the deutocerebral olfactory and accessory lobes as the major output pathway of these two neuropils (reviews [25,57,58]). In the crayfish *Procambarus clarkii*, the projection neurons may amount to at least 100,000 per hemi brain [85]. The layout of this neural pathway as well as physiological aspects have been thoroughly analyzed in crayfish, lobsters, and spiny lobsters [58,69,71,73,79,85-91] as well as a recent set of experiments applying focal injections of lipophilic tracers by Sullivan and Beltz [82,92-94]. The paired olfactory globular tracts emerge medially from the olfactory lobes and approach the midline of the brain where they meet to form a chiasm (located slightly dorsal to the central body) and finally target the lateral protocerebrum.

Details of the projection neuron pathway are best understood in crayfish by far, in which the hemiellipsoid body is divided into two distinct lobes, neuropil regions I and II, which are composed of thousands of microglomeruli. The terminal branches of the projection neuron tract from their ***olfactory ***lobe extend bilaterally to the medulla terminalis (*Procambarus clarkii; *[58,85,93]) or to the medulla terminalis and the hemiellipsoid body region I (*Cherax destructor; *[94]). The projection neuron tract from the ***accessory ***lobe bifurcates in the chiasm and targets the hemiellipsoid body region II on both sides of the brain [93,94]. Within the microglomeruli, projection neuron axons terminate within endings termed rosettes, each of which makes as many as 165 output synapses upon local interneurons [85,86]. The thousands of local protocerebral interneurons associated with the crayfish hemiellipsoid body [86,88,89,95] respond to olfactory stimulation of the antennae I, stimulation of tactile receptors innervating the antennae II, and photic stimulation of the eyes [89-91]. Because the local interneurons associated with the crayfish accessory lobe provide tactile and visual sensitivity as well as chemosensory input, the projection neuron pathway from the accessory lobe is thought to take a central role in conveying some of these stimuli to the hemielliposid body [94]. Thus, the lateral protocerebrum is thought to be a higher integration center for chemosensory, mechanosensory and visual stimuli [25,57,58,69,92,93,96,97].

In addition to several reptantian decapods including the spiny lobster *Panulirus argus *[63], several crayfish species (see above), and the American lobster *Homarus americanus *[92,93], comprehensive information on the lateral protocerebrum architecture obtained with methods that are comparable to ours is available for representatives of the non-reptantian malacostracan taxa Stomatopoda, Dendrobranchiata, Caridea, and Stenopodidea [82]. As mentioned above, in lobsters and crayfish, the projection neuron pathway associated with the accessory lobe (multi-modal stimuli) projects exclusively to the hemiellipsoid body whereas the projection neuron pathway associated with the olfactory lobe (chemosensory stimuli) projects mostly to the medulla terminalis [92,93]. Accessory lobes are thought to have emerged as an apomorphy of the Reptantia [40,98]. In non-reptantian crustaceans (which lack accessory lobes) the olfactory globular tract is the output pathway of the olfactory lobe alone and terminates both in the medulla terminalis and the hemielliposid body. Therefore, Sullivan and Beltz [82] wanted to know if in non-Reptantia the hemiellipsoid body may function primarily as second-order olfactory neuropil and wanted to trace the changes in the relative importance of the medulla terminalis *versus *hemiellipsoid body in the olfactory pathway during evolution of the Malacostraca. These authors found that although the specific targets of the olfactory globular tract have been conserved, the relative extent to which this tract innervates the medulla terminalis *versus *the hemiellipsoid body can nevertheless vary markedly between species so that the relative importance of these two neuropils within the olfactory pathway has changed. More specifically, in the ground pattern of the Eumalacostraca, the medulla terminalis was the most important second order olfactory neuropil but this role gradually shifted more towards the hemiellipsoid body in the evolutionary trajectory towards the Eureptantia [82]. The evolutionary appearance of the accessory lobe in the Reptantia then initiated new changes of the connectivity between the lateral protocerebrum in that the input from the olfactory lobe to the medulla terminalis was maintained but the hemiellipsoid body attained a new, dominant multi-modal input from the accessory lobe.

Anomura and Brachyura are considered to be among the most highly derived decapod taxa [99], and the accessory lobes have become largely reduced in these two groups [40,52]. Sandeman and Scholtz [98] consider this reduction to be a synapomorphy of these two taxa. Not much detailed information is available about the lateral protocerebrum in Anomura and Brachyura that may serve as a comparison to the findings presented here [52]. However, from the former study it is clear that another representative of the Coenobitidae, the giant robber crab *Birgus latro*, also has an extremely enlarged hemiellipsoid body that matches the size of the olfactory lobe (Fig. 11 in [52]), similar to the situation in *Coenobita clypeatus*. A recent re-investigation of *B. latro *has confirmed this finding [84]. Both the genera *Birgus *and *Coenobita *have evolved a sense of aerial olfaction that is highly relevant for their behavior and display large olfactory lobes compared to other Crustacea. The members of these two taxa may have compensated for their small accessory lobe by enlarging the hemiellipsoid body in order to maintain good analyzing capacities for olfactory stimuli.

As *C. clypeatus*, the *B. latro *hemielliposid body also displays a lamellar organization [52,84]. As elaborated above, the hemiellipsoid neuropil in crayfish and lobsters is not lamellar but organized into thousands of microglomeruli. Nevertheless, in the American lobster (Homarida; [92]) and the mantis shrimp *Gonodactylus bredini *(Stomatopoda) the hemiellipsoid is composed of a hemielliptical, concave sheet of neuropil, the "cap", that surrounds an inner neuropil "core" from which it separated by an intermediate layer [82]. This architecture resembles the situation in *C. clypeatus *with the exception that here, a second core neuropil is present. The lateral protocerebra of Dendrobranchiata, Caridea, and Stenopodidea, in contrast, display either poorly differentiated hemiellipsoid bodies or architectures that are different from the cap/core motif [82]. One explanation could be that the cap/core arrangement characterizes the ground pattern of Malacostraca, to become reduced or modified in multiple ways during the evolution of this taxon. A more common motif is the layering of the hemiellipsoid body neuropil. Such layers, although very few in numbers are present in representatives of the Stomatopoda, the Caridea, and the Stenopodidea [82]. In a preliminary study of the hemiellipsoid body in the marine hermit *Pagurus bernhardus*, we also found a moderate number of layers (Harzsch and Hansson, unpublished data). Therefore, we suggest that layers in the hemielliposid body may characterize the malacostracan ground pattern whereas the microglomeruli in Astacida and Homarida are derived. In this view, the lamellar architecture in the hemiellipsoid body of Coenobitidae may be an elaboration of the ancestral "layer motif" and mirror the enlarged olfactory lobes and the massif input of olfactory projection neurons. Interestingly, the vertical and medial lobes of insect secondary olfactory neuropils, the mushroom bodies, also display a layered structure in some species (reviews [100,101]). The architecture of these layers has been thoroughly studies in the honey bee [102-104] and in the cockroach [105-107] in which also the developmental emergence of the layers has been explored [108,109]. Emerging evidence suggests that ancestral insect mushroom bodies were composed only of the pedunculus and the lobes but lacked a calyx [101,110], but it is unclear if the lobes had a layered structure in the insect ground pattern. More detailed analyses with additional markers will be necessary to explore the question if the laminar structure in the hemiellipsoid bodies of Coenobitidae evolved convergently to that in the insects or if this design goes back to a shared principle in the common ancestor of Crustacea and Hexapoda.

### Comparison to other Crustacea – the optic neuropils

In malacostracan crustaceans, the visual input from the compound eyes is processed in three columnar optic neuropils, the lamina, the medulla, and the lobula the architecture of which is best understood in crayfish [111-114]. For a comparison with brachyuran crabs, which are the sister taxon to the Anomura [44], a recent paper by Sztarker and co-workers [61] on *Chasmagnatus granulatus *is most relevant as well as other recent papers that have explored evolutionary aspects of crustacean optic neuropils [60,106,115].

In Crustacea, the axons of the histaminergic retinal photoreceptors R1–R7 project the eye receptor mosaic retinotopically onto the lamina ([114]; R8 terminates in the medulla). From the lamina, the retinotopic mosaic is projected onto the medulla which in crayfish is divided into an outer and an inner neuropil [114]. The neurochemical architecture of the medulla is diverse (discussed in [79]). The processes of tachykinin-related peptide immunoreactive neurons are arranged in four horizontals layers within the crayfish medulla [116]. Crustacean-SIFamide immunoreactivity is localized in many columnar elements within the outer neuropil as well as the inner neuropil of the crayfish medulla [79]. Serotonergic neurons are associated with the medulla of Mysidacea [117] and crayfish and in the latter group the serotonin immunoreactive neurites branch in three horizontal layers of the medulla [72,118,119]. Overall, the arrangement of the serotonergic cell somata in a lateral group and a second group distal to the medulla as well as the horizontal layering that we observed in *C. clypeatus *is quite similar to that in these other crustaceans. The three distinct layers of FaRP immunoreactive neurites in the *C. clypeatus *medulla have a close parallel in the crayfish where three SIFamide immunoreactive horizontal strata are present [79].

The third optic neuropil of Malacostraca, the lobula, (traditionally called "medulla interna"; see e.g. [63]) is the most proximal neuropil to show a clear-cut columnar and stratified organization. In crayfish, afferents from the medulla that target the lobula comprise bundles of retinotopic columnar relay neurons and columnar T-neurons [114]. With histological techniques, seven main strata can be recognized in the crayfish lobula, three of which receive input from the medulla [114]. This complicated system of horizontal layers is also apparent in immunohistochemical studies [79,116]. In the present report, we could distinguish as many as fourteen different layers in a double labeling experiment of synaptic proteins and FaRP immunoreactive material. Sztarker and co-workers [61], using Bodian's reduced silver method, observed four strata of tangential processes in transverse sections of the lobula in the brachyuran crab *Chasmagnatus granulatus*. Some of these strata contain the dendritic trees of wide-field motion-sensitive neurons. The strata are separated by several layers that consist of terminal processes of columnar elements and by tangential elements that extend orthogonally [61]. In Fig. 7A and 7C of their contribution, at least twelve or more layers can be distinguished in transverse sections of the lobula but due to the incompatible methodology it is not possible to relate these layers to our own findings. In summary, we conclude that, at the coarse level of comparison with other studies that is possible at this time, the visual system in *C. clypeatus *in many aspects is similar to that of other Malacostraca considering its architecture and neurochemistry.

## Conclusion

The primary olfactory centers are the dominating neuropils of the medial brain in *C. clypeatus*, which parallels behavioral findings of an excellent sense of aerial olfaction in these animals. The secondary olfactory centers (hemiellipsoid bodies) are also large and organized into parallel neuropil lamellae. Future studies using backfill methods should analyze more details of the olfactory pathway in these animals specifically with respect to comparing the hemiellipsoid body architecture in *C. clypeatus *to the lamellate structure of the vertical and medial lobes in insect mushroom bodies. The organization of the optic neuropils and those neuropils associated with antenna 2 suggest that *C. clypeatus *has visual and mechanosensory skills that are comparable to those of other Decapoda. Preliminary studies on another highly terrestrial group of Crustacea, the Oniscoidea ("wood lice"; members of the Isopoda), suggest that, contrary to the Coenobitidae, the deutocerebral olfactory pathway does not play a significant role for aerial olfaction in these animals [36-38]. Future studies on terrestrial Amphipoda, Astacida, and Brachyura may shed light on how frequently the establishment of an aerial sense of olfaction evolved in Crustacea during the transition from sea to land.

## Methods

### Immunohistochemistry

Adult specimens of *Coenobita clypeatus *(Herbst, 1791; Anomura, Coenobitidae) were obtained from the "Zoologischer Großhandel Peter Hoch" (August Jeanmaire Str. 12, 79183 Waldkirch, Germany). The animals (ca. 5–8 cm total length) were anaesthetized for at least one hour on ice and then their brains were dissected in phosphate buffered saline (0.1 M PBS, pH 7.4). The isolated brains and eyestalks were fixed overnight in 4% PFA in 0.1 M PBS, ph 7.4 at 4°C. After fixation the tissues were washed for 4 hours in several changes of PBS and subsequently sectioned (80 μm) with a HM 650 V vibratome (Microm). Overnight permeabilization in PBTx (0.3% Tx-100 in 0.1 M PBS, pH 7.4) at 4°C the specimens was followed by incubation in the primary antibodies overnight at 4°C. The antisera that we used were: polyclonal rabbit anti FMRFamide (1:1000; DiaSorin, Cat. No. 20091, Lot No. 923602); polyclonal rabbit anti-serotonin (1:2000; ImmunoStar Incorporated, Cat. No. 20080, Lot No. 541016); monoclonal mouse anti-synapsin „SYNORF1“ antibody (1:30 in PBS-TX, [120] antibody provided by E. Buchner, Universität Würzburg, Germany); monoclonal mouse anti-glutamine synthetase (1:100; BD Biosciences Pharmingen, Cat. No. 610517). After incubation in the primary antisera, tissues were washed in several changes of PBS for 4 hours at room temperature and incubated in secondary Alexa Fluor488 or Alexa Fluor 546 IgGs (1:50, Invitrogen, Eugene, Oregon, USA) overnight at 4°C. All sections were routinely counterstained with the nuclear dye bisbenzimide (0.1%, Hoechst H 33258) for 15 min. at room temperature. Some sections were processed with a histochemical counter stain, a high-affinity probe for actin, by adding Phallotoxins conjugated to Alexa Fluor 546 (Molecular Probes; concentration 200 units/ml) to the secondary antibody in a dilution 1:50. Finally, the tissues were washed for at least 2 h in several changes of PBS and mounted in GelMount (Sigma).

We carried out three sets of triple labeling experiments i.e. combinations of markers:

1. synapsin immunolocalization with histochemical counter stains of actin and cell nuclei.

2. RFamide-like immunoreactivity combined with synapsin immunolocalization and nuclear counter stain

3. glutamine synthetase-like immunoreactivity (GSir) combined with serotonin immunolocalization (5-HTir) and nuclear counter stain.

The localization of synapsin and actin as general markers of neuropils structures was chosen to provide a good overview over the general brain architecture. Immunohistochemistry against RFamide-like peptides and against serotonin labels subsets of neurons and allows the visualization of the neuronal processes. Furthermore, these two markers have been applied in a wide range of crustaceans thus allowing a good interspecific comparison. The anti-glutamine synthetase is a glia marker and was chosen to also include non-neuronal elements of the nervous system into this analysis. The nuclear marker HOECHST was used to show the localization of the various cell clusters. Thus, the chosen markers complement each other and the whole set is well suited to visualize a broad range of different structures thus providing a detailed insight into the crab's brain anatomy. Our analysis is based on more than 5 successfully processed brains per marker set, and the labeling pattern was consistent between these specimens. The specimens were viewed with a Zeiss AxioImager equipped with the Zeiss Apotome structured illumination device for optical sectioning ("grid projection"). Digital images were processed with the Zeiss AxioVision software package. In addition, specimens were analyzed with the laser scanning microscope Zeiss LSM 510 Meta. Double-labeled specimens were generally analyzed in the multi-track mode in which the two lasers operate sequentially, and narrow band-pass filters were used to assure a clean separation of the labels and to avoid any crosstalk between the channels. All images were processed in Adobe Photoshop using global picture enhancement features (brightness/contrast).

### Specificity of the antisera

The tetrapeptide FMRFamide and FMRFamide-related peptides (FaRPs) are widely distributed among invertebrates and vertebrates and form a large neuropeptide family with more than 50 members all of which share the RFamide motif (reviews: [121-127]). In malacostracan Crustacea, at least twelve FaRPs have been identified and sequenced from crabs, shrimps, lobsters and crayfish [128,129]. These peptides range from seven to twelve amino acids in length and most of them share the carboxy terminal sequence LRFamide. The antiserum we used was generated in rabbit against synthetic FMRFamide (Phe-Met-Arg-Phe-NH2) conjugated to bovine thyroglobulin (DiaSorin, Cat. No. 20091, Lot No. 923602). According to the manufacturer, staining with this antiserum is completely eliminated by pretreatment of the diluted antibody with 100 μg/ml of FMRFamide. We repeated this experiment and preincubated the antiserum with 100 μg/ml FMRFamide (Sigma; 16 h, 4°C) and this preincubation abolished all staining. Because the crustacean FaRPs know so far all share the carboxy terminal sequence LRFamide we conclude that the DiaSorin antiserum that we used most likely labels any peptide terminating with the sequence RFamide. Therefore, we will refer to the labeled structures in our specimens as "RFamide-like immunoreactive (RFir) neurons" throughout the paper.

The antiserum against serotonin (ImmunoStar Incorporated; Cat. No. 20080, Lot No. 541016) is a polyclonal rabbit antiserum raised against serotonin coupled to bovine serum albumin (BSA) with paraformaldehyde. The antiserum was quality control tested by the manufacturer using standard immunohistochemical methods. According to the manufacturer, staining with the antiserum was completely eliminated by pretreatment of the diluted antibody with 25 μg of serotonin coupled to BSA per ml of the diluted antibody. We repeated this control with the serotonin-BSA conjugate that was used for generation of the antiserum as provided by ImmunoStar (Cat. No. 20081, Lot No. 750256; 50 μg of lyophilized serotonin creatinine sulfate coupled to BSA with paraformaldehyde). Preadsorption of the antibody in working dilution with the serotonin-BSA conjugate at a final conjugate concentration of 10 μg/ml at 4°C for 24 h completely blocked all immunolabelling. We performed an additional control and preadsorbed the diluted antiserum with 10 mg/ml BSA for 4 h at room temperature. This preadsorption did not affect the staining, thus, providing evidence that the antiserum does not recognize the carrier molecule alone. The manufacturer also examined the cross reactivity of the antiserum. According to the data sheet, with 5 μg, 10 μg, and 25 μg amounts, the following substances did not react with the antiserum diluted to 1:20,000 using the horse radish peroxidase (HRP) labeling method: 5-hydroxytryptophan, 5-hydroxyindole-3-acetic acid, and dopamine.

**Figure 20:**
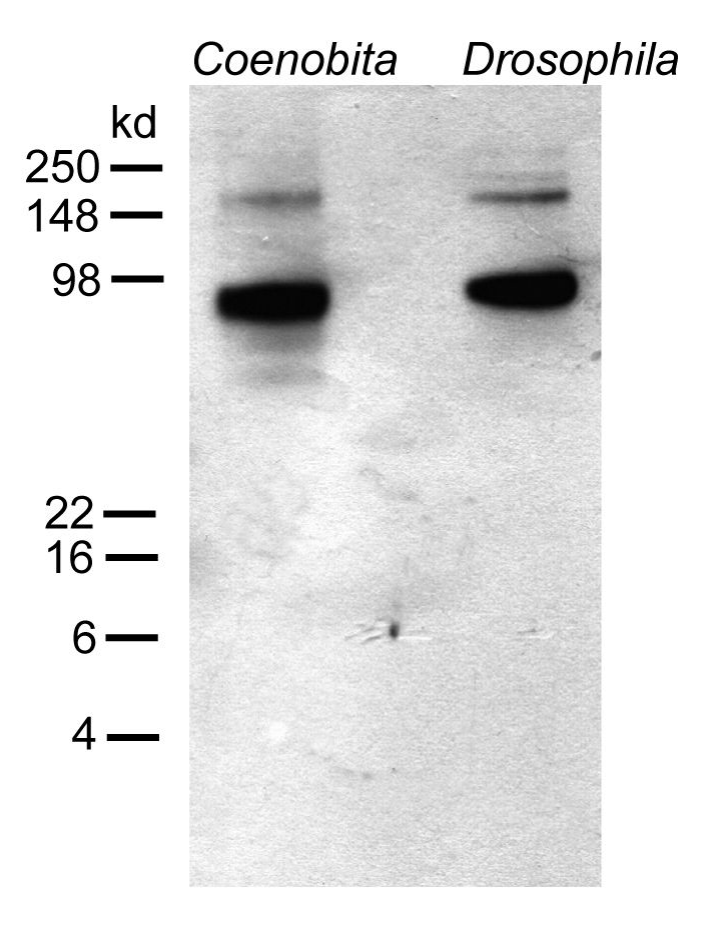
**Western Blot analysis of the SYNORF1 antibody (anti-synapsin) comparing brain tissue of *Drosophila *and *Coenobita*.** The antibody provided identical results for both species staining one strong band around 80–90 kDa and a second weaker band slightly above 148 kDa. This pattern closely resembles the results obtained in the original publication in which the antibody was characterized for *Drosophila *(Klagges et al. 1996).

The monoclonal mouse anti-*Drosophila *synapsin „SYNORF1“ antibody (provided by E. Buchner, Universität Würzburg, Germany) was raised against a *Drosophila *GST-synapsin fusion protein and recognizes at least four synapsin isoforms (ca. 70, 74, 80, and 143 kDa) in western blots of *Drosophila *head homogenates [120]. In western blot analysis of crayfish homogenates, this antibody stains a single band at ca. 75 kDa (see [130]). We conducted a western blot analysis comparing brain tissue of *Drosophila *and *Coenobita*. The antibody provided identical results for both species staining one strong band around 80–90 kDa and a second weaker band slightly above 148 kDa (Fig. 20). Our analysis strongly suggests that the epitope which SYNORF 1 recognizes is strongly conserved between the fruit fly and the hermit crab. Similar to *Drosophila*, the antibody consistently labels brain structures in representatives of all major subgroups of the malacostracan crustaceans [42,131-134] in a pattern that is consistent with the assumption that this antibody does in fact label synaptic neuropil in Crustacea. In the crustacean first optic neuropil (the lamina), synapsin labeling is weak compared to the other brain neuropils ([131]; and present report). Similarly, in *Drosophila *labeling of the lamina is weak because photoreceptors R1–R6 which have their synapses in the lamina contain very little of the presently known synapsin homolog isoforms [120]. The antibody also labels neuromuscular synapses both in *Drosophila *and in Crustacea [131]. These close parallels in the labeling pattern of SYNORF1 between *Drosophila *and various Crustacea strengthens the claim that it also recognizes crustacean synapsin homologs. This antibody even labels synaptic neuropil in an ancestral clade of protostomes, the Chaetognatha [135] suggesting that the epitope that this antiserum recognizes is conserved over wide evolutionary distances.

The monoclonal mouse anti-glutamine synthetase antibody (1:100; BD Biosciences Pharmingen, Cat. No. 610517) was generated using sheep glutamine synthetase, an octamer of identical 45 kDa subunits, as the immunogen. According to the manufacturer, this antibody labels a single 45 kDa in Western blot analysis of rat brain homogenates. In Western blots of crayfish (*Procambarus clarkii*) brain homogenates, the antibody labels a single band at ca. 44 kDa (see [130]) which is in the same range as the glutamine synthetase in the spiny lobster *Panulirus argus *(42 kDa; [55]) suggesting that the antibody that we used also binds to crustacean glutamine synthetase. Because we did not conduct a western blot analysis in *C. clypeatus*, we will refer to the labeled structures in our specimens as "glutamine synthetase-like immunoreactivity" (GSir) throughout the paper.

In additional control experiments for possible nonspecific binding of the secondary antiserum, we omitted the primary antiserum, replaced it with blocking solution, and followed the labeling protocol as above. In these control experiments, staining was absent.

### 3D reconstruction

Image stacks obtained from z-series by the Zeiss LSM 510 Meta were directly loaded into the 3D reconstruction software Amira (Mercury Systems) operated on a Fujitsu Siemens Celsius 560 workstation. The surface reconstructions in Figs. 9 and 13 were generated by using Amira's "wrap" module for semiautomatic segmentation.

### Neuroanatomical nomenclature

We describe our data in the context of crayfish brain anatomy as laid out in the studies of Blaustein and co-workers [63], Sandeman et al. [40,52], Sandeman and Scholtz [98] and Sandeman and Mellon [57]. Sandeman and co-workers [52] have compared the neuroanatomical nomenclature used during the past and have suggested a standard nomenclature for the components of the brain of Decapoda which is adopted here with minor modifications that concern the optic ganglia [114]. Sandeman and co-workers [52] have also recognized 17 different clusters of cell bodies associated with the crayfish brain, which they examined and numbered, 1–17, from anterior to posterior. We will adhere to this nomenclature and refer to cell clusters by their given numbers in parentheses.

## Abbreviations

**Optic ganglia**. ICh inner optic chiasm. La Lamina (lamina ganglionaris). Me Medulla (medulla externa). OCh outer optic chiasm. **Lateral protocerebrum**. Cap cap neuropil of the hemiellipsoid body. CO1, CO2 core neuropils 1 and 2 of the hemiellipsoid body. HN hemiellipsoid body. IL1, IL2 intermediate layers 1 and 2 of the hemiellipsoid bodyLo Lobula (medulla interna). LoP Lobula "plate". MT Medulla terminalis **Median Protocerebrum**. AMPN anterior medial protocerebral neuropil. PMPN posterior medial protocerebral neuropil. CB central body. PB protocerebral bridge. PT protocerebral tract. X chiasm of the olfactory globular tract **Deutocerebrum**. A1Nv nerve of antenna 1. AcN acessory lobe/neuropil. CA cerebral artery. LAN lateral antenna 1 neuropil. MAN median antenna 1 neuropil. mF median foramen. ncON non-columnar olfactroy neuropil. OGT olfactory globular tract. OGTN olfactory globular tract neuropil. OGTNa accessory olfactory globular tract neuropil. ON olfactory lobe/neuropil. pF posterior foramen. VC ventral neuropil column. **Tritocerebrum**. A2Nv nerve of Antenna 2. AnN antenna 2 neuropil. CEC circumesophageal connectives

## Authors' contributions

SH designed this study, carried out the immunohistochemical experiments and microscopic analysis and drafted the manuscript. BSH conceived the study, and participated in its design and coordination and helped to draft the manuscript. Both authors read and approved the final manuscript.
